# Regulating Interfacial Microenvironment in Aqueous Electrolyte via a N_2_ Filtering Membrane for Efficient Electrochemical Ammonia Synthesis

**DOI:** 10.1002/advs.202309200

**Published:** 2024-05-10

**Authors:** Mengdi Liu, Yan Ma, Sai Zhang, Min Chen, Limin Wu

**Affiliations:** ^1^ Department of Materials Science and State Key Laboratory of Molecular Engineering of Polymers Fudan University Shanghai 200433 China

**Keywords:** ammonia synthesis, N_2_ filtering membrane, N_2_ rich microenvironment, nitrogen reduction reaction, polydimethylsiloxane

## Abstract

Electrochemical synthesis of ammonia (NH_3_) in aqueous electrolyte has long been suffered from poor nitrogen (N_2_) supply owing to its low solubility and sluggish diffusion kinetics. Therefore, creating a N_2_ rich microenvironment around catalyst surface may potentially improve the efficiency of nitrogen reduction reaction (NRR). Herein, a delicately designed N_2_ filtering membrane consisted of polydimethylsiloxane is covered on catalyst surface via superspreading. Because this membrane let the dissolved N_2_ molecules be accessible to the catalyst but block excess water, the designed N_2_ rich microenvironment over catalyst leads to an optimized Faradaic efficiency of 39.4% and an NH_3_ yield rate of 109.2 µg h^−1^ mg^−1^, which is superior to those of the most report metal‐based catalysts for electrochemical NRR. This study offers alternative strategy for enhancing NRR performance.

## Graphical abstract

A delicately designed N_2_ filtering membrane consisted of polydimethylsiloxane is covered on catalyst surface via superspreading. The N_2_ rich environment is generated via filtering and accumulating dissolved N_2_ molecules around catalyst while blocking excess water. It leads to an optimized NRR performance of 39.4% FE and an NH_3_ yield rate of 109.2 µg h^−1^ mg^−1^.

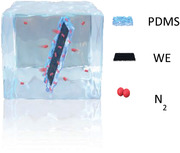

## 1 Introduction

Ammonia (NH_3_) has long been considered more than an essential chemical for fertilizer production, its potential as energy carrier in electricity generation, roads, maritime and air transportation has drawn enormous attention.^[^ ^1^ ^]^ Conventional ammonia synthesis through Haber‐Bosch technology fed with hydrogen (H_2_) from natural gas consumes nearly 2% of global energy expansion and creates 3%–5% annual carbon dioxide (CO_2_) emission.^[^ ^2^ ^]^ Among green ammonia synthesis technologies, electrochemical hydrogenation of nitrogen gas (N_2_) with water (H_2_O) stands out due to its environmental friendly, sustainable and cost‐effective features.^[^ ^3^ ^]^ However, increasing the electrocatalytic production of NH_3_ suffers from two main obstacles: low Faradaic efficiency (FE) and poor yield rate.^[^ ^4^ ^]^ Strong N≡N triple bond (941 kJ mol^−1^), low solubility of N_2_ in aqueous electrolyte and advantageous hydrogen evolution reaction (HER) limit the practical application of electrochemical nitrogen reduction reaction (NRR).^[^ ^5^ ^]^ Numbers of efficient catalysts have been developed to enhance the NRR performance through improving the ability of N_2_ activation or exposing more active sites for NRR process, yet the crucial influence of N_2_ feed in electrolytes has somehow been overlooked.^[^ ^6^ ^]^ Only minute amount of N_2_ could dissolve in aqueous electrolyte and even less can reach the catalyst surface caused by the slow diffusion rate, leading to the unsatisfactory NRR efficiency.^[^ ^7^ ^]^ Additionally, the great amount of protic water easily covers the majority of catalyst surface making HER predominate which further impedes the NRR process.^[^ ^8^ ^]^ Therefore, the improvement of N_2_ supply offers promising opportunity to enhance the NRR performance.^[^ ^9^ ^]^

Increasing the N_2_ partial pressure has been considered as a valid strategy of concentrating N_2_ in electrolytes, yet higher pressure needs stronger reactors and causes more waste of unreacted N_2_.^[^ ^6^, ^10^ ^]^ On the other hand, non‐aqueous electrolytes offer another approach to improve NRR efficiency since N_2_ has higher solubility in organic solvents.^[^ ^11^ ^]^ Unfortunately, the utilization of organics generates harmful pollutants and usually requires more energy input, not mentioning the limited choices of suitable proton source.^[^ ^12^ ^]^ Thus, proper technics or methods to efficiently enhance the N_2_ concentration around catalyst surfaces in aqueous electrolyte under ambient conditions are desirable.^[^ ^13^ ^]^ Polymer membranes allowing sufficient N_2_ penetration with suitable amount of protons due to their different permeability of N_2_ and H_2_O offers great chance to enhance NRR performance.^[^ ^5^, ^14^ ^]^ Polydimethylsiloxane (PDMS) has been widely applied for gas separation in food industry, it can potentially rationalize the N_2_ concentration over catalyst surface while prohibiting excess H_2_O aggregation.^[^ ^15^ ^]^ Delicate synthesis of PDMS can be achieved through superspreading strategy since the N_2_/H_2_O permeability significantly depend on the thickness of the membrane.^[^ ^16^ ^]^ Ideally, it is believed to improve both selectivity and NH_3_ yield rate if the NRR favorable microenvironment around catalyst is obtained attribute to the N_2_ accumulation.^[^ ^17^ ^]^

Herein, the Fe single atom was anchored on nitrogen doped carbon framework as nitrogen reduction catalyst followed with covering with PDMS membrane. Because PDMS allows the dissolved N_2_ passing and aggregating around catalyst surfaces while less H_2_O molecules do so, which further suppresses HER side reaction and improves NRR efficiency. Accordingly, 39.4% faradaic efficiency along with 109.2 µg h^−1^ mg^−1^ NH_3_ yield rate can be achieved under −0.6 V versus RHE in 0.1 M Na_2_SO_4_, which is more than three times that without PDMS membrane. This study offers a convenient and universal strategy to regulate the microenvironment over catalyst for enhancing the efficiency of NH_3_ synthesis in aqueous system.

## 2 Results and Discussion

### 2.1 Synthesis and Characterization of Catalysts

**Figure 1:**
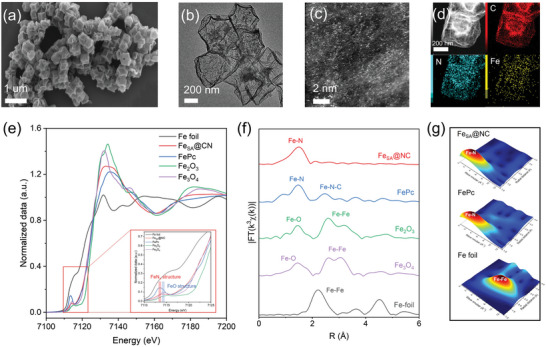
a) SEM image, b) TEM image, c) HAADF‐STEM image and d) EDS mapping of Fe_SA_@NC. e) Fe K‐edge XSNES and f) FT EXAFS of Fe_SA_@NC and reference samples. g) WT EXAFS contour plots of Fe K‐edge for Fe_SA_@NC and reference samples.

Owing to the unique catalytic reactivity and high atomic utilization, Fe single atom catalyst was synthesized as follows: First, ZIF‐8 was prepared under room temperature by simply mixing Zn(NO_3_)_2_ with 2‐methylimidazole in methanol following by loaded with dopamine hydrochloride (PDA) and Fe(NO_3_)_3_. Th precursor (Fe‐PDA@ZIF‐8) was then pyrolyzed under 920 °C for 2 h in Ar to obtain final product denoted as Fe_SA_@NC.^[^ ^18^, ^19^ ^]^ The ZIF‐8 particles present uniform cubic morphology as well as Fe‐PDA@ZIF‐8 (Figure S1, Supporting Information). After loaded with Fe source and PDA, obvious particles are observed on the surfaces of ZIF‐8 cubes. From X‐ray diffraction patterns (XRD, Figure S2, Supporting Information), only broad and weak peaks can be identified after calcination indicating the carbonization of ZIF‐8 substrate, the absence of metal Fe or Fe(NO_3_)_3_ peaks suggests the possibility of successful synthesis of Fe single atoms. After pyrolysis of Fe‐PDA@ZIF‐8, two Raman peaks assigned to D and G band at 1346 and 1583 cm^−1^, respectively, can be observed in both Fe_SA_@NC and nitrogen doped carbon sample (NC) (Figure S3, Supporting Information).^[^ ^20^ ^]^ **Figure** **1**a,b exhibit the scanning electron microscopy (SEM) and transmitting electron microscopy (TEM) images of Fe_SA_@NC sample. Post‐thermal treatment generates hollow nanocubes with a diameter of ≈400 to 500 nm, no distinguishable particles are found around the surface of catalyst. High angle annual dark field scanning TEM (HAADF‐STEM) was utilized to further characterize the as‐prepared catalyst. As shown in Figure 1c, uniformly dispersed bright spots illustrate that the Fe species succeed in forming single atoms on the carbon nanocubes without obvious aggregation of the metal species observed. Energy dispersive spectroscopy (EDS) in Figure 1d confirms the existence and homogeneous dispersion of Fe element over the substate. X‐ray photoelectron spectra (XPS) of Fe_SA_@NC and bare carbon substrate indicate peaks attributed to graphitic N, pyrrolic N and pyridinic N are observed in both samples after deconvolution of N 1s spectrum (Figure S4, Supporting Information). However, an additional Fe‐N peak appearing in Fe_SA_@NC which implies that the N element has been successfully doped and coordinated with the Fe species. It can barely find any changes in C 1s spectrum between Fe_SA_@NC and NC sample, indicating the similar structure of carbon framework. Although the intensity of XPS for Fe 2p in Fe_SA_@NC is relatively weak due to the small amount of Fe (Figure S5, Supporting Information), it could still confirm the existence of Fe element in as‐prepared sample. The accurate Fe content is ≈6.8 wt.% (Figure S6 and Table S1, Supporting Information) loaded on the substrate determined through inductively coupled plasma emission spectrometer (ICP).

In order to unveil more detailed information about the chemical environment and atomic structure of as‐prepared catalyst, X‐ray adsorption near‐edge structure (XANES) and extended X‐ray adsorption fine structure (EXAFS) analysis were conducted. According to the energy of adsorption edge (Figure 1e), Fe_SA_@NC shows similar curve with iron phthalocyanine (FePc) and Fe_2_O_3_, indicating an oxidation state between +2 and +3. Moreover, the inset picture clearly shows an almost identical pre‐edge peak at ≈7113.2 eV in Fe_SA_@NC and FePc, implying the FeN_x_ structure in Fe_SA_@NC.^[^ ^21^ ^]^ In contrast, other pre‐edge peaks of Fe‐O located at ≈7114.2 eV appear in Fe_2_O_3_ and Fe_3_O_4_, respectively. By analyzing the results of EXAFS (Figure 1f), only one main peak at 1.5 Å appears in Fe_SA_@NC which is attributed to first coordination shell of Fe‐N and similar to FePc reference. Fe‐Fe peaks at 2.57, 2.57, and 2.2 Å in Fe_2_O_3_, Fe_3_O_4_ and Fe foil, respectively, are not found in as‐prepared sample that further indicates the atomic distribution of Fe species.

Wavelet transform investigation in Figure 1g verifies the above discussion. Similar intensity maximum at ≈2.2 Å^−1^ in Fe_SA_@NC and FePc which quite differs from that obtained from Fe foil (≈6.7 Å^−1^) confirms the FeN_4_ structure.^[^ ^22^ ^]^ The EXAFS fitting data in Figure S7 and Table S2 (Supporting Information) suggest that the experimental line of Fe species in Fe_SA_@NC matches well with the simulation data when the parameters were set with a bond length of 2.0 Å and a coordination number of 3.7, which is in line with XANES and WT‐EXAFS results. All aforementioned information suggests the uniform dispersion of Fe single atoms on nitrogen doped hollow carbon nanocubes.

**Figure 2:**
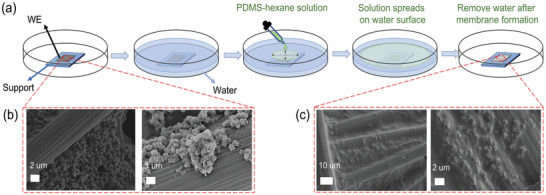
a) Schematic illustration of PDMS formation on WE via superspreading. b,c) SEM images of WE before and after PDMS covering, respectively.

Afterwards, Fe_SA_@NC was loaded on carbon paper as working electrode (WE) covered by PDMS membrane on both sides through superspreading (denoted as Fe_SA_@NC‐Px, x stands for the amount of PDMS‐hexane solution used during synthesis). **Figure** **2**a depicts the schematic illustration of PDMS formation. Clear stripes of carbon nanofibers and the edges of the nanocubes can be observed in Figure 2b before PDMS formation. In contrast, the as‐prepared nanocubes are definitely buried by a thin film which blurs the edges and corners of loaded catalyst (Figure 2c). Fourier Transform Infra‐Red (FTIR) spectrum indicates that obvious peak of Si‐O‐Si symmetric vibration is located at ≈800 cm^−1^ on Glass sample while no significant signal appears for carbon paper loaded with catalyst (Figure S8, Supporting Information). After covered with PDMS membrane, new peaks belonged to PDMS arise between 500 to 1500 cm^−1^, for instance, 1259 cm^−1^ for Si‐CH_3_ bending and 1015 cm^−1^ for Si‐O‐Si stretching.^[^ ^23^ ^]^ The carbon paper with PDMS showed almost identical spectrum compared to glass sample covered with PDMS which undoubtably confirms the formation of designed membrane on WE. The thickness of PDMS was manipulated via adjusting the amount of PDMS‐hexane solution during superspreading. As shown in Figure S9 (Supporting Information), the thickness increase from 40 to 200 nm with the increasing usage of the precursor solution.

### 2.2 Electrocatalytic NRR Performances

**Figure 3:**
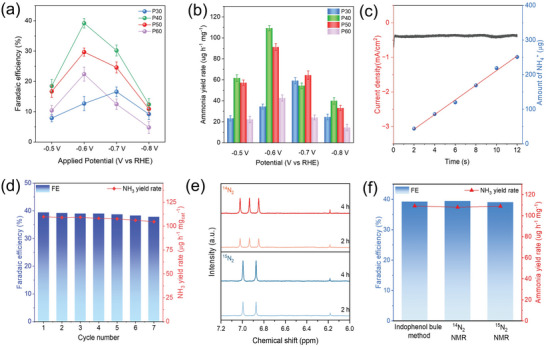
a,b) FE and NH_3_ yield rate of Fe_SA_@NC‐Px samples in 0.1 M Na_2_SO_4_. c) Long‐term test of Fe_SA_@NC‐P40 in 0.1 M Na_2_SO_4_ under −0.6 V versus RHE and corresponding amount of generated NH_3_. d) Reproducibility of Fe_SA_@NC‐P40 in 0.1 M Na_2_SO_4_ under −0.6 V versus RHE. e) ^1^H NMR spectrum of electrolytes after NRR process using ^14^N_2_ and ^15^N_2_. f). Comparison of FE and corresponding NH_3_ yield rate measured by NMR and indophenol blue method.

The NRR performance of as‐prepared samples was then evaluated via a three electrodes system in an H‐cell in 0.1 M Na_2_SO_4_. We first investigated the intrinsic catalytic ability of Fe_SA_@NC sample. Linear sweep voltammetry curve (LSV) suggests a slightly higher current density in N_2_ atmosphere under the potential range of −0.2 to −0.5 V versus RHE (Figure S10, Supporting Information). The FE and NH_3_ yield rates were measured by indophenol blue method and shown in Figures S11 and S12 (Supporting Information). Fe_SA_@NC obtains a 12.4% faradaic efficiency and an NH_3_ yield rate of 44.3 µg h^−1^ mg^−1^ which are unsatisfactory. Next, the samples covered with PDMS were investigated. LSV curves suggested that total current densities of samples with PDMS were decreased compared with Fe_SA_@NC which will be discussed in later section (Figure S13, Supporting Information). The thicker the PDMS membrane, the smaller the overall current density is. Both of FE and NH_3_ yield rate increase first then decrease with the applied potential increase due to the severe HER side reaction under higher potentials (**Figure** **3**a,b). Notably, all samples with PDMS show better NRR efficiency compared with bare Fe_SA_@NC, inferring the positive effect of PDMS membrane. Among them, Fe_SA_@NC‐P40 obtained an optimized FE of 39.4% along with an NH_3_ yield rate of 109.2 µg h^−1^ mg^−1^ which is superior to most of the recent reported metal‐based catalysts (Table S3, Supporting Information). The turnover numbers (TON) of as‐prepared catalysts are listed in Table S4 (Supporting Information), in which Fe_SA_@NC‐P40 shows the best TON which is in line with the electrochemical performance measurements. A relatively steady current density can be observed during 12 h electrolysis over Fe_SA_@NC‐P40 sample (Figure 3c). Additionally, the steady chronoamperometry curves of the sample at various potential shown in Figure S14 (Supporting Information) indicate the stability of Fe_SA_@NC‐P40. The chemical environment and valence state of Fe species remain unchanged compared with fresh catalyst as presented by XANES and EXAFS in Figure S15 (Supporting Information). No obvious peaks of Fe‐Fe or Fe‐O are found meaning no aggregation or oxidation of the loaded Fe single atoms. Almost identical XRD and XPS data (Figures S16 and S17, Supporting Information) before and after electrolysis agree with the XAFS results indicating the as‐prepared catalyst is highly stable under electrochemical test. Moreover, the corresponding amount of generated NH_3_ shows a well fitted linear relationship with reaction time, suggesting that the NH_3_ was produced from N_2_ reduction. Limited performance loss could be found in the cycling test, indicating the high stability of as‐prepared sample (Figure 3d). After electrolysis, FTIR plots and SEM images show that the PDMS membrane remain intact which further confirms the stability of as‐prepared catalyst (Figures S18 and S19, Supporting Information).

In order to decipher the NH_3_ source, various and systematic control experiments were carried out (Figure S20, Supporting Information). At first, N_2_ feed gas was replaced by Ar and only trace amount of NH_3_ could be detected which ruled out the possibility of catalyst decomposition. Then the catalyst was immersed in N_2_ saturated 0.1 M Na_2_SO_4_ under OCP, it can hardly generate any NH_3_, indicating no exterior NH_3_ pollution.^[^ ^24^ ^]^ Since the NO_x_ and NH_3_ in feeding gas are important error sources, we checked the existence of these species in feeding gas and electrolytes. NO_x_ and NH_3_ were first examined via spectrophotometric method.^[^ ^25^ ^]^ Figures S21 and S22a (Supporting Information) suggested no NO_x_ species in ^14^N_2_, ^15^N_2_ feeding gas and Na_2_SO_4_ electrolyte. Indophenol bule detection (Figure S22b, Supporting Information) of NH_3_ further exclude the possibility of NH_3_ in feeding gas and electrolyte. Mass spectrometer (MS) was further utilized to evaluate the purity of gas sources. Figure S23 (Supporting Information) shows that the major peaks located at 28 and 30 are attributed to ^14^N_2_ and ^15^N_2_, respectively, while no molecular pieces of NO_x_ species exist which is consistent with spectrophotometric method. Notably, the peaks at 28 for ^14^N_2_, 32 for O_2_ and 44 for CO_2_ in Figure S23b (Supporting Information) are caused by unavoidable air leakage during sampling process which also happened for the test of ^14^N_2_. Other possible interference, like existence of NO_2_^−^ in electrolyte, is also inspected via an UV‐Vis spectrometer. As illustrated in Figures S24 and S25 (Supporting Information), NO_2_^−^ is undetectable either before or after the electrolysis. Therefore, the catalyst reduces N_2_ to NH_3_ only under the co‐existence of N_2_ feed gas and certain applied potential. To more accurately track the N source, isotope labelling experiments were subsequently performed. The electrolysis was taken using purified ^14^N_2_ and ^15^N_2_ and the corresponding ^1^H NMR of the electrolytes is illustrated in Figure 3e. The generated NH_3_ amount for both ^14^N_2_ and ^15^N_2_ were increased over reaction time and perfectly lay on the calibration curves, respectively (Figures S26 and S27, Supporting Information).^[^ ^3^, ^26^ ^]^ The calculated FE and yield rate via NMR also agree with those measured by indophenol blue method (Figure 3f). Then side product like N_2_H_4_ is detected by Watt and Chrisp method (Figures S28 and S29, Supporting Information), it suggests that NH_3_ is the major N_2_ reduction product during the electrochemical process and the remaining Faradaic efficiency is attributed to HER side reaction along which is calculated by gas chromatography (GC) (Figure S30, Supporting Information). Above results clearly confirm that the NH_3_ is produced by the electrocatalytic reduction of N_2_ feed gas.

### 2.3 Origin of the High NRR Performance

To reveal the origin of the performance enhancement, the ability of PDMS to adjust the concentration of N_2_ and H_2_O in interfacial microenvironment over catalyst surface was investigated both experimentally and theoretically. At first, the hydrophobic character of PDMS was examined via contact angle experiment. As illustrated in Figure S31 (Supporting Information), the contact angle of water droplet on glass dramatically increases from 74.4 ° to 121.4 ° before and after the covering of PDMS, indicating the significant water resistance of the poly membrane.^[^ ^27^ ^]^ Due to the crossed fiber structure and micro‐structure of loaded catalyst, WE without PDMS also exhibits a hydrophobic character. Notably, after loading of PDMS, samples show better hydrophobicity. However, the thickness of PDMS has negligible influence on the contact angle. It is reported that the thickness of PDMS has a significant effect on the permeation of H_2_O molecules, therefore the mass difference method was utilized to further analyze the correlation between the water permeability and thickness.^[^ ^15b^ ^]^ PDMS membranes with various thickness were formed on water surface and settled for 24 h under same conditions. Then the lost mass of water can be utilized to evaluate the H_2_O permeability of PDMS. As shown in Table S5 (Supporting Information), the thicker the PDMS membrane, the less the water lost is, indicating that H_2_O permeability decreases as the thickness increases. It can be implied that PDMS on catalyst inhibits water permeation. Less water reaching on catalyst surfaces means lower possibility of HER side reaction during NRR process. It well explains the decreased total current densities in LSV curves of the samples covered with PDMS in Figure S13 (Supporting Information). We then analyzed the electrochemical impedance spectroscopy (EIS) of as‐prepared samples. Figure S32 (Supporting Information) indicates that thicker PDMS films bring larger charge transfer resistances (R_ct_) which also leads to the decreased total current density. In return, the inferior HER performance could be achieved and resulted in enhanced NRR performance. It is a trade‐off between the suppression of HER and overall reactivity which means a suitable thickness of PDMS could obtain an acceptable R_ct_ and a relatively high NRR efficiency. Abovementioned results clearly suggest that PDMS has certain ability to reduce the water content over catalyst surface and further mitigate the impact of HER side reaction.

**Figure 4:**
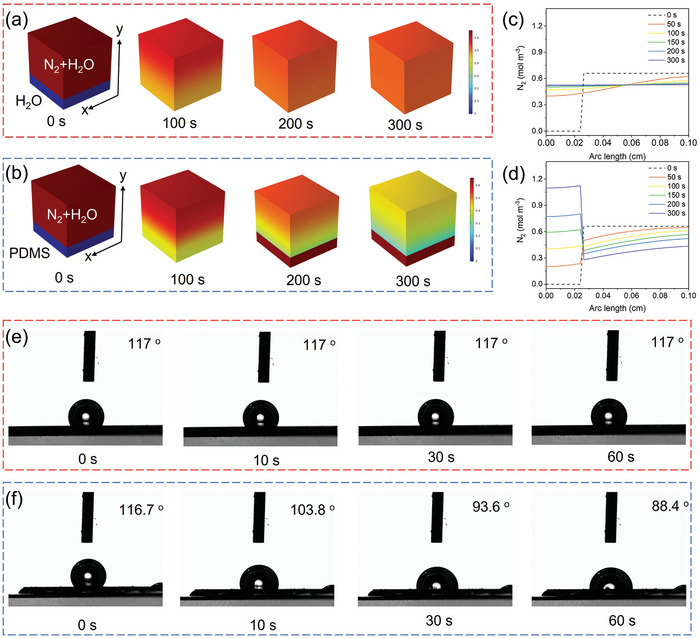
a) Snapshots of N_2_ diffusion in a box with pure water and, b) PDMS at the bottom as time progressed. c,d) The corresponding N_2_ concentration versus y‐direction at different time. e) Optical images of N_2_ bubble on working electrode without and, f) with PDMS over time.

On the other hand, the dynamic equilibrium of N_2_ gas was investigated via finite element simulation (FES). Cubic boxes with a diameter of 0.1 cm for FES analysis were created and divided into two parts in which the upper part was filled with N_2_ saturated H_2_O and the lower one fifth of box is filled with pure water (**Figure** **4**a) or pure PDMS (Figure 4b).^[^ ^13^ ^]^ The dissolved N_2_ molecules slowly move to the bottom and finally reaches an almost uniform concentration in the box which was only filled with water. Corresponding curves of N_2_ concentration versus y‐direction further confirm the free diffusion owing to the concentration gradient (Figure 4c). In contrast, PDMS extracts the N_2_ from water over time and eventually forms a region with much higher N_2_ concentration. Figure 4d clearly illustrates that N_2_ molecules at the interface of H_2_O and PDMS migrate into the lower part first, then accumulate attributing to the higher solubility of N_2_ in PDMS.^[^ ^28^ ^]^ To get more accurate information of N_2_ dynamics in designed catalytic system, a similar box was separated by a 100 nm PDMS membrane where the upper part was filled with H_2_O and N_2_, yet only H_2_O in the lower part (Figure S33b, Supporting Information).^[^ ^29^ ^]^ Undetectable difference could be observed by comparing the results of snapshots of the simulation systems with or without the PDMS membrane, however the corresponding curves of N_2_ concentration along the y‐direction showed interesting results (Figure S34, Supporting Information). The N_2_ concentration near the left side of the PDMS membrane became higher compared with pure water over time which can be implied that N_2_ was first quickly got into PDMS, then passed through it and got into the pure water region (Figure S34c and S34d, Supporting Information). Therefore, concentration of N_2_ at the bottom after the application of PDMS membrane was slightly larger than the pure water system which can be clearly verified by the enlarged plots. This means a more effective N_2_ diffusion process could occur by penetrating a thin PDMS membrane. Combined with previous results, it is believed that dissolved N_2_ can accumulate inside the PDMS then pass through it to reach the catalyst surface for further NRR reaction.

To gain more visualized information of the enhancement of N_2_ diffusion through PDMS membrane, the contact angle experiments of N_2_ bubbles over different samples were carried out. Figure 4e,f display the optical images of the behavior of N_2_ bubbles on samples with and without PDMS membrane over time. A N_2_ bubble with 2 µL volume was applied to the surface of a normal WE, no significant difference could be detected as time passed. In contrast, the N_2_ bubble obviously shrank on the WE with PDMS membrane within 60 s, clearly confirming the improved N_2_ diffusion through PDMS.

**Figure 5:**
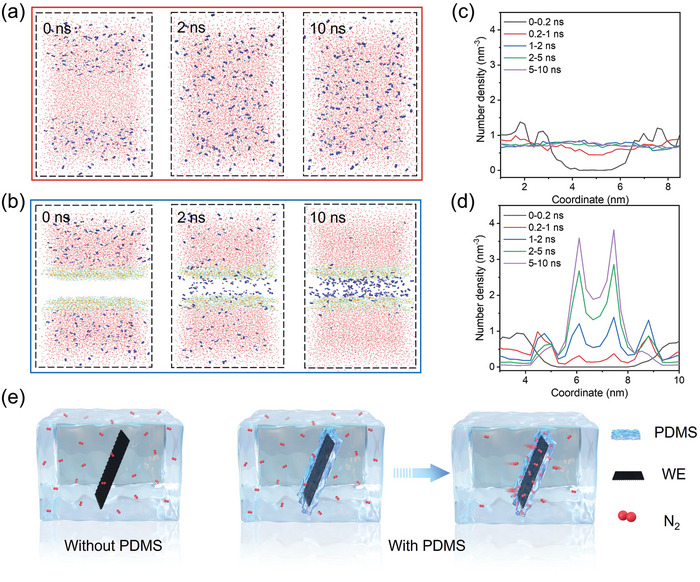
a) MD simulation of N_2_ dynamics for pure water and, b) with PDMS membrane. c,d) Corresponding N_2_ density along y‐direction. e) Schematic illustration of N_2_ diffusion over working electrode with and without PDMS.

For further theoretical investigation, the N_2_ diffusion was studied via molecular dynamics (MD). The first system was created by applying N_2_ molecules in pure water (**Figure** **5**a) while two PDMS thin films were applied to the second system (Figure 5b) to imitate the working electrode covered with PDMS. N_2_ molecules reached a uniform dispersion in the first system which agrees with the FES result. However, N_2_ clearly aggregates in PDMS and further pass through it and accumulates at the middle of the two membranes in Figure 5b. The corresponding concentration of N_2_ in above mentioned systems versus y‐direction clearly suggest that N_2_ could migrate though the PDMS and concentrated inside the membrane which creates a N_2_ dominated microenvironment around the catalyst surface (Figure 5c,d). These results well match with the FES simulation and contact angle experiments. Figure S35 (Supporting Information) shows van der Waals’ force among N_2_, H_2_O and PDMS in above mentioned systems. The energy between N_2_ and H_2_O remained unchanged in pure water system which implies a uniform dispersion of N_2_ in water. However, an obvious strong interaction between N_2_ and PDMS can be indicated by the increase of the energy which evidently suggests that N_2_ can aggregate in PDMS. Additionally, corresponding H_2_O distribution in two systems are also presented in Figure S36 (Supporting Information), indicating the good capability of PDMS for impeding H_2_O penetration in accordance with the results of H_2_O contact angle experiments. According to computational study and experimental evidences, the N_2_ diffusion behavior was depicted in Figure 5e. Dissolved N_2_ molecules will randomly dispersed in the electrolyte and only limited amount of them can reach the catalyst surface which leads to poor NRR reactivity. In contrast, after covered with PDMS membrane on the working electrode, N_2_ will be filtrated to aggregate inside the membrane while prohibiting most of the H_2_O to get close to the catalyst. A N_2_ rich microenvironment will be generated around the catalyst which makes a nitrogen reduction preferred surrounding and leads to a much better NRR performance.

### 2.4 DFT Calculations and Electrocatalysis Mechanism

**Figure 6:**
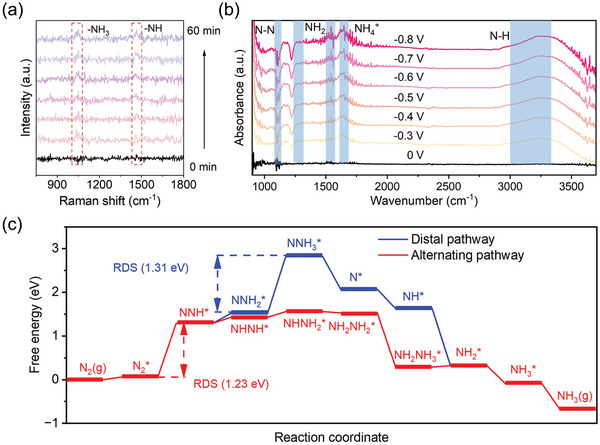
a) Time‐resolved Raman spectrum of Fe_SA_@NC in 0.1 M Na_2_SO_4_ under −0.6 V versus RHE. b) In‐situ FTIR spectrum of Fe_SA_@NC in 0.1 M Na_2_SO_4_ under different applied potential. c) Free energy diagrams of NRR process over Fe_SA_@NC.

Time‐resolved Raman measurements and in‐situ FTIR spectrometry were carried out to investigate the NRR process over as‐prepared catalyst. As shown in **Figure** **6**a, two Raman peaks assigned to NH_3_ and ‐NH appear at ≈1056 and 1489 cm^−1^ as time involved, respectively, indicating the occurrence of nitrogen reduction on the catalyst.^[^ ^30^ ^]^ Furthermore, the in‐situ FTIR peaks located at 1270, 1623 and 3230 cm^−1^ which are attributed to ‐NH_2_ wagging, NH_4_^+^ vibration and NH stretching, respectively, were clearly observed with the applied potential increase in Figure 6b.^[^ ^31^ ^]^ Notably, the inversed peak assigned to ‐N‐N stretching at 1097 cm^−1^ indicating the consumption of N_2_ molecules on the catalyst. These results clearly suggest that the nitrogen can be effectively reduced over as‐prepared catalyst. The structure property and NRR reaction mechanisms were studied by density functional theory (DFT). The adsorption models over different FeN_4_ sites were analyzed. XPS data suggested that multiple types of N existed in carbon framework, namely pyrrolic N, pyridinic N and graphitic N. Normally, Fe atoms connected to pyrrolic N or pyridinic N were considered as catalytic active sites, thus several possible adsorption candidates are constructed and presented in Figure S37 (Supporting Information).^[^ ^18^, ^32^ ^]^ The lowest formation energy was obtained when N_2_ adsorbs on FeN_4_‐pyridinic N site through end‐on mode, thus this structure was further applied to the DFT study. As illustrated in Figure 6c, the free energy diagrams of NRR process over Fe_SA_@NC for both alternating pathway and distal pathway were investigated. The first hydrogenation step for N_2_ molecules (N_2_* to NNH*) plays as the rate‐determining step (RDS) for alternating pathway which gains a free energy change of 1.23 eV. A higher energy of 1.31 eV was obtained for NNH_2_* to NNH_3_* as RDS in distal mechanism. Therefore, above experimental and theoretical results suggest that NH_3_ can be efficiently generated over as‐prepared catalyst through alternating pathway (Figure S38, Supporting Information).

## 3 Conclusion

In summary, we have demonstrated a N_2_ favorable filtering membrane consist of PDMS to improve the selectivity and reactivity of ammonia synthesis in aqueous electrolyte. The PDMS membrane effectively attracts the dissolved N_2_ molecules to aggregate around catalyst surface while prohibiting excess water accumulation owing to its different permeability of N_2_ and H_2_O. Accordingly, The Fe_SA_@NC‐P40 exhibits an optimized NRR efficiency of 39.4% FE and 109.2 µg h^−1^ mg^−1^ ammonia yield rate with relatively high stability in 0.1 M Na_2_SO_4_. This study highlights the importance of N_2_ supply in ammonia synthesis and provide a promising strategy to regulate the reactant material for enhancing the NRR efficiency.

## Conflict of Interest

The authors declare no conflict of interest.

## Supporting information

Supporting Information

## Data Availability

The data that support the findings of this study are available from the corresponding author upon reasonable request.
