# Supplementary material for: Regulating Interfacial Microenvironment in Aqueous Electrolyte via a N2 Filtering Membrane for Efficient Electrochemical Ammonia Synthesis
Source: Adv Sci (Weinh). 2024 May 10;11(28):2309200. doi: 10.1002/advs.202309200 (PMC11267261; doi:10.1002/advs.202309200)
Supplement: Supplementary file 1 — Supporting Information [file ADVS-11-2309200-s001.pdf]

ADVANCED SCIENCE
Open Access

Supporting Information

for *Adv. Sci.*, DOI 10.1002/advs.202309200

Regulating Interfacial Microenvironment in Aqueous Electrolyte via a N_2 Filtering Membrane for Efficient Electrochemical Ammonia Synthesis

*Mengdi Liu, Yan Ma, Sai Zhang, Min Chen and Limin Wu**

Regulating interfacial microenvironment in aqueous electrolyte via a N_2 filtering membrane for efficient electrochemical ammonia synthesis

*Mengdi Liu, Yan Ma, Sai Zhang, Min Chen, Limin Wu**

Department of Materials Science and State Key Laboratory of Molecular Engineering of Polymers, Fudan University, Shanghai, 200433, China

Corresponding E-mail: lmw@fudan.edu.cn

Experimental Section

Chemicals: Zinc nitrate hexahydrate ($Zn(NO_3)_2 \cdot 6H_2O$, 99%), Iron(III) nitrate nonahydrate ($Fe(NO_3)_3 \cdot 9H_2O$, 99.9%), 2-methylimidazole ($C_4H_6N_2$, 99%), potassium hydroxide (KOH, 99%), tris(hydroxymethyl)aminomethane ($NH_2C(CH_2OH)_3$, 99%), dopamine hydrochloride ($(HO)_2C_6H_3CH_2CH_2NH_2 \cdot HCl$, 99%), hydrazine ($NH_2NH_2$ H_2O, 24-26% in H_2O) ammonia chloride (NH_4Cl, 99.998%), Nafion perfluorinated resin solution (5 wt% in lower aliphatic alcohols and water, contains 15–20% water) and urea ($NH(CO)_2$, 99%) were purchased from Sigma-Aldrich. Polydimethylsiloxane (PDMS, SYLGARD 184) was received from Dow Corning Corp. Sodium sulphate (Na_2SO_4, 99%), melamine ($C_3H_6N_6$, 99%), sodium nitroferricyanide ($Na_2[Fe(CN)_5NO] \cdot 2H_2O$, 99.98%) were purchased from Aladdin Industrial Corporation. Sodium hypochlorite (NaClO, 5% available chlorine), p-dimethylaminobenzaldehyde ($C_9H_{11}NO$, 98%), N-(1-naphthyl) ethylenediamine dihydrochloride ($C_{12}H_{14}N_2$, 98%) and *p*-aminobenzenesulfonamide ($C_6H_8N_2O_2S$, 99%) were purchased from J&K Scientific. Dimethyl sulfoxide-d6 (DMSO-*d*6) was purchased

from Cambridge Isotope Laboratories. Inc. Salicylic acid ($C_7H_6O_3$, 99.5%) was purchased from Macklin Biochemical Technology Co., Ltd.

Synthesis of ZIF-8 nanocubes: 10 g of 2-methylimidazole was dissolved in 100 mL methanol. Then 4.7 g $Zn(NO_3)_2 \cdot 6H_2O$ was dissolved in another 100 mL methanol and poured into the above solution under sonification. The mixed solution was stirred for 24 h under room temperature. As synthesized ZIF-8 nanocubes were washed with methanol and dried in oven.[1]

Synthesis of Fe_{SA}@NC: 0.2 g ZIF-8 and 0.12 g tris(hydroxymethyl)aminomethane were dissolved in 100 mL deionized water and ethanol solution (volume ratio 1:1). Then 60.6 mg $Fe(NO_3)_3 \cdot 9H_2O$ and 28.4 mg dopamine hydrochloride were dissolved in 100 mL deionized water. Fe solution was poured into ZIF-8 solution and stirred for 4 h under room temperature. The product was washed with ethanol and water (Fe-PDA@ZIF-8). Next, equivalent amount of Fe-PDA@ZIF-8 and melamine were mixed together and placed in a ceramic crucible. The mixture was heated to 920 ^{o}C at a rate of 5 ^{o}C min^{-1} for 2 h under Ar.[1] After cooling to room temperature, the carbonized powder was washed with 3 M H_2SO_4 for 12 h. The final product (Fe_{SA}@NC) was washed with water and dried in oven for further use. Bare nitrogen doped carbon sample was synthesized through the above-mentioned procedure without adding Fe precursor and named as NC.

Synthesis of PDMS membrane on working electrode: First, PDMS base solution was prepared by mixing PDMS and curing agent with a mass ratio of 10:1. Then the base solution was added into equivalent hexane as precursor. The working electrode (WE) was prepared as follow: 2 mg Fe_{SA}@NC was dispersed in 120 μL Nafion solution and 880 μL ethanol. The mixture was sonicated for 1 h to make it homogeneous. Finally, 40 μL of the

solution was drop-added on a carbon paper with 1 x 1 cm^2 area. Then the PDMS membrane was covered on as-prepared working electrode in a homemade device. Typically, the working electrode was placed on a support in a petri dish, then certain amount of water was added to make the liquid surface 3 mm higher than the working electrode. Various volume of PDMS-hexane precursor was dropped on water surface. After curing for 3 days, water was removed and formed PDMS membrane was covered on the top side of WE. The procedure was repeated to cover the other side of the WE to get the final product (Fe_{SA}@NC-Px, where x stands for the volume of PDMS-hexane solution).

Material characterization: The scanning electron microscopy (SEM) images and transmitting electron microscopy (TEM) images were collected using a field emission scanning electron microscope (Zeiss, Ultra 55, 15 kV) and a transmitting electron microscope (FEI, Tecnai G2 20 TWIN, 200 kV), respectively. An aberration-corrected EM-ARM300F (JEOL, 200 kV) was utilized to obtain the scanning transmission electron microscopy (STEM) images. Powder X-ray diffraction (XRD) was investigated using a D8 Advance diffractometer (Bruker) with Cu Kα radiation λ = 1.5406 A. Fourier Transform Infra-Red (FTIR) data was obtained on Nicolet IS50 spectrometer (Thermofisher Scientific). XPS spectra was collected by a scanning X-ray microprobe (ULVAC-PHI, PHI 5000 C and PHI 5300), using C 1 s (284.6 eV) as a reference. Contact angle experiments were measured by a TBU 95 contact goniometer (Dataphysics, German). Fe contents were measured by an Agilent 5110 inductively coupled plasma-optical emission spectrometer (ICP-OES). Fe K-edge analysis was performed with Si(111) crystal monochromators at the BL11B beamlines at the Shanghai Synchrotron Radiation Facility (SSRF) (Shanghai, China). The XAFS spectra were recorded at room temperature using a 4-channel Silicon

Drift Detector (SDD) Bruker 5040. Negligible changes in the line-shape and peak position of both Fe K-edge XANES spectra were observed between two scans taken for a specific sample. The XAFS spectra of these standard samples (Fe foil, FePc, Fe_2O_3 and Fe_3O_4) were recorded in transmission mode. The spectra were processed and analyzed by the software codes Athena and Artemis.

Electrochemical measurement: The electrochemical experiments were carried out in an H-cell using a three electrodes system in 0.1 M Na_2SO_4 with a WaveDrive2000 electrochemical workstation (Pine, United States). The H-cell was separated with a DuPont 117 proton exchange membrane where working electrode and AgCl reference electrode were placed in cathodic chamber and Pt counter electrode was placed in anodic chamber. The N_2 feed gas was purified with 0.1 M H_2SO_4 to remove any possible external NH_3 followed with 0.1 M KOH to get rid of other N-containing pollutants and acid. At last, the molecular sieves were used to eliminate remaining impurities and H_2O. The purified feed gas was purged into the cathodic chamber for 30 min before any electrochemical measurements and kept purging during the test. The linear sweep voltammetry curves were carried out with a scanning rate of 10 mV s^{-1}. Electrochemical impedance spectroscopy (EIS) was carried out with an AC amplitude of 10 mV and frequency range from 0.1 Hz to 1 MHz. Chronoamperometry plots were measured for 60 min. The following Nernst equation was used to convert the potentials to the reversible hydrogen electrode (RHE):

$$E_{RHE} = E_{Ag/AgCl} + E_{Applied\ potential} + 0.059pH$$

A PHS-3C pH meter (Shanghai Leici Instrument Co., China) was used for the test of pH of the electrolytes.

Ammonia quantification: The produced NH_3 was determined with Indophenol blue method and ^1H NMR method. The indophenol blue method was as follow: 2 mL NH_4Cl standard solution was mixed with 2 mL NaOH solution (1 M) containing salicylic acid (5 wt%) and sodium citrate (5 wt%). Next, 1 mL NaClO solution (0.05 M) and 0.2 mL sodium nitroferricyanide solution (1 wt%) were mixed with the above solution and aged for 1 h at room temperature. Then the adsorption of the solutions at 655 nm wavelength was measured with an UV-vis spectrophotometer (HATACHI, U4100). The obtained data was used to plot the calibration curve of absorbance-concentration. The produced NH_3 in each NRR experiment was measured by the same procedure. For NMR method, the standard solution was prepared by mixing 0.5 mL NH_4Cl as reference sample, 0.1 mL dimethyl sulfoxide-*d*6 (DMSO-*d*6) as solvent, 0.1 mL HCl (0.1 M) and certain amount of maleic acid as internal standard. As-prepared standards were measured by a Avance NEO 600 NMR spectrometer (Bruker). The concentration of NH_4^+ and peak ratio between NH_4^+ and maleic acid was plotted as calibration curve. The electrolytes after electrolysis were used to replace the standards with the same method to quantitatively determine NH_3 produced.

The Faradaic efficiency was calculated using the following equation:

$$FE = 3F \text{ x } C_{NH_3} \; x \; V/(18Q)$$

where F is the Faraday constant (96485 C mol^{-1}), C_{NH3} is the NH_3 concentration (g L^{-1}), V is the volume of electrolyte (L), and Q is the total charge (C).

The NH_3 yield was obtained as follows:

$$R_{NH_3} = C_{NH_3} \text{ x } V/(m_{cat.} \text{ x } t)$$

where t is the reduction time (h) and $m_{cat.}$ is the mass of catalyst loaded on working electrode (mg).

Isotope labelling experiment: $^{15}N_2$ was utilized to accurately track the N element during the electrochemical process. First, the $^{15}N_2$ gas was pre-treated with 1 M H_2SO_4 and 1 M KOH to remove any impurities or exterior NH_3 pollutant. Then it was purged into the reaction chamber before electrolysis for 20 min and kept purging during test. The ^{1}H NMR of the electrolyte after reaction was carried out with the same procedure described in previous section. $^{15}N_2$ was also replaced with $^{14}N_2$ for comparison.

NO_x detection: NO_x contaminates were identified via spectrophotometric method and Mass spectrometer (MS). For spectrophotometric method, 0.5 g sulfanilic acid and 5 mL acetic acid were dissolved in 90 mL DI water followed with the addition of 5 mg *N*-(-1-naphthyl)-ethylenediamine dihydrochloride.[2] The color reagent was then prepared by mixing above mentioned solution with water by a volume ration of 1:4. The tested gas was first fed into an oxidation tube than passed through the color reagent for a period of time. The calibration curve was plotted using potassium nitrite as standard. For MS characterization, 50 μL feeding gas was transferred using a syringe and analyzed via a GCMS-QP2020 NX (SHIMADZU).

N_2H_4 quantification: The N_2H_4 was determined by The Watt and Chrisp method. Typically, 5.99 g of p-dimethylaminobenzaldehyde was dissolved in HCl (concentrated, 30 mL) and ethanol (300 mL) to get color reagent. Then 5 mL of test solution were mixed with the above solution. A UV-Vis spectrophotometer was used to measure the absorbance at 455 nm of the mixed solution after aging for 20 min at 25 ^{o}C. Certain amount of N_2H_4 was mixed with 0.1 M Na_2SO_4 as standards, then the absorbance of the standard solutions was measured and used for plotting the concentration-absorbance calibration curve.

NO_2^- quantification: First, 0.2 g of N-(1-naphthyl) ethylenediamine dihydrochloride, 4 g of *p*-aminobenzenesulfonamide, and 10 mL of phosphoric acid ($\rho = 1.685$ g mL^{-1}) was mixed in 50 mL deionized water and stored at 4 ^{o}C for further use. Then 5 mL of sample solution was combined with 0.1 mL of abovementioned solution and aged for 20 min at room temperature. The absorbance at 540 nm was spectrophotometrically measured and used for calibration curve. The electrolytes were further tested using the same procedure to quantitatively determine the NO_2^- content.

In situ characterization: The in situ Raman characterization was carried out using a tailor-made cell on HORIBA, XploRa spectrometer. Ag/AgCl reference electrode and Pt counter electrode were placed in anodic chamber which is separated to cathodic chamber containing working electrode by a Nafion 117 membrane. The electrochemical cell was filled with N_2 saturated electrolyte before test. The spectrum was taken under -0.6 V vs RHE at different time. The in situ FTIR measurements were conducted using Attenuated Total Reflectance (ATR) model on Thermo Scientific, Nicolet iS50 spectrometer. The Si ATR crystal is placed on the side of a customized spectro-electrochemical cell. As-prepared catalyst was deposited on a polycrystalline Au film as working electrode, Ag/AgCl and Pt were used as reference electrode and counter electrode, respectively. All spectrums were obtained with 8 cm^{-1} resolution for 64 scans using the FTIR spectrometer.

Computational details: All spin-polarized density functional theories (DFT) were performed by using the Vienna Ab initio Simulation Package (VASP).[3] The Perdew-Burke-Ernzerhof generalized-gradient approximation functional was used to describe the interaction between electrons.[4] The energy cutoff was set to 400 eV. The calculation for pyridinic and pyrrolic structures were carried out using the 3x3x1 and 4x4x1 Gamma-

Centered k-points grids. The vacuum region was set to be 15 Å in z direction to prevent the interaction between two adjacent surfaces. The energy criterion was set to 10^{-5} eV.

The adsorption energies (ΔG) were obtained by

$$\Delta G = \Delta E_{DFT} + \Delta ZPE - T\Delta S$$

where ΔE_{DFT} is the reaction energy calculated from DFT; ΔZPE is the zero-point energy; ΔS is the change in entropy.[5]

The finite element simulation (FES) was carried out using COMSOL Multiphysics software coupled with the transport of the diluted species module. A cubic box with a diameter of 0.1 cm was constructed and was divided into two parts. In the initial FES model, the upper section with 0.08 cm length in the y direction is filled with H_2O and N_2 molecules based on the actual N_2 solubility, and the lower section with 0.02 cm length is filled with pure H_2O. Then, the simulations for PDMS and PDMS membrane are performed by using PDMS to replace pure water in the lower part of the box and using 100 nm PDMS membrane to separate the upper and lower part, respectively. FES runs of 300 s were conducted for the models, respectively.

Molecular dynamics (MD) simulations are performed with Gromacs version 2019.6.[6] In the system with PDMS, two PDMS films are separated by 2 nm, and each film consists of 100 PDMS chains with a thickness of 1 nm. The outer region of the PDMS films is solvated by a liquid phase containing 10787 water molecules and 250 N_2 molecules. In the control system, PDMS films are eliminated for comparison. Above model construction procedures are completed with PACKMOL software.[7] TIP3P model is adopted for water.[8] PDMS chain is modeled by the general amber force field.[9] N_2 is represented by the TraPPE model.[10] Coulombic interactions are computed with the particle-mesh Ewald method. Van

der Waals interactions are computed by the Lennard Jones (LJ) potential which is truncated at 1.2 nm, and LJ parameters between different atoms are determined by Lorentz-Berthelot mixing rules. Energy-minimization is conducted for initial configurations. Subsequently, 50-ns production runs are conducted at 298.15 K and 1 bar. The motion equations are integrated by leapfrog algorithm. A time step of 1 fs is used. Three-dimensional periodic boundary conditions are applied during all simulations.

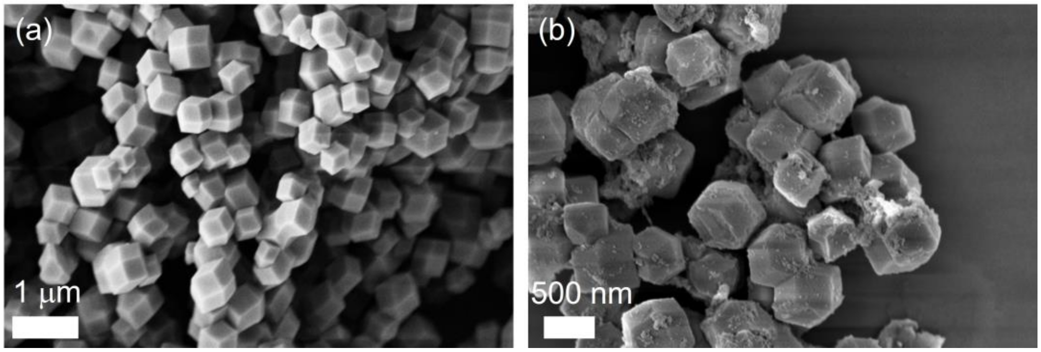

Figure S1. SEM images of (a) ZIF-8 and(b) Fe-PDA@ZIF-8.

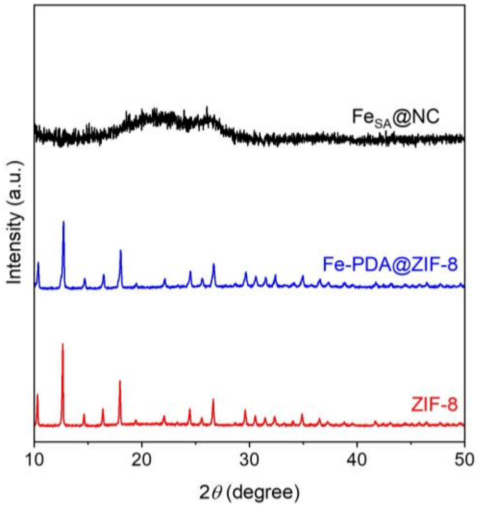

Figure S2. XRD patterns of ZIF-8, Fe-PDA@ZIF-8 and Fe_{SA}@NC.

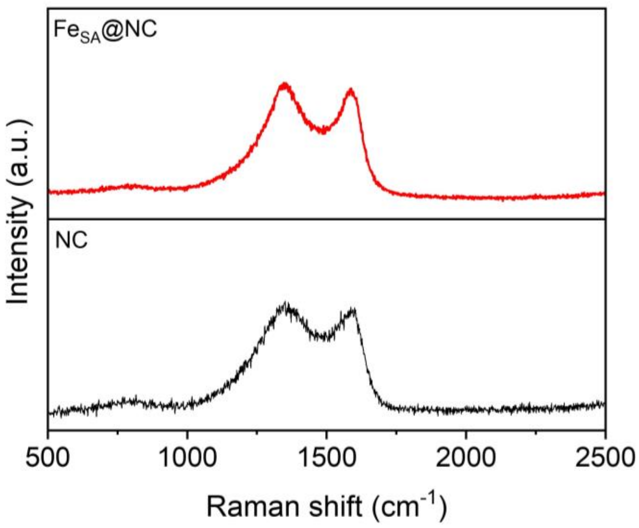

Figure S3. Raman spectrum of NC and Fe$_{SA}$@NC.

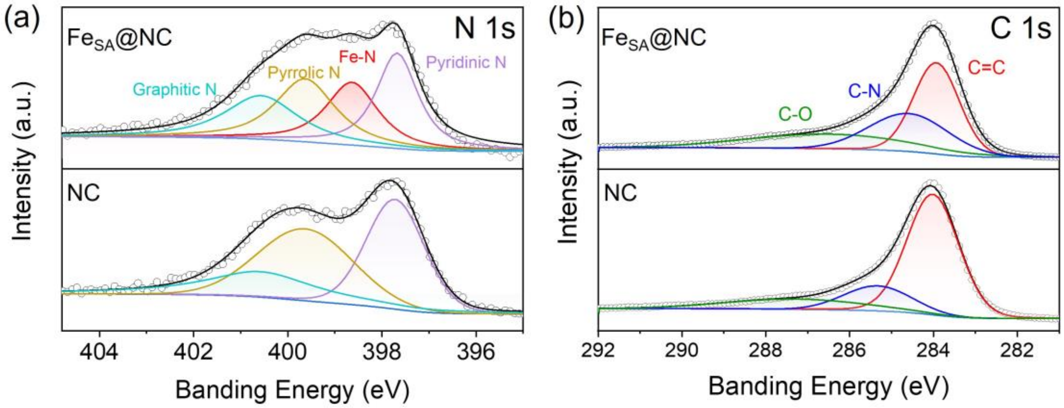

Figure S4. (a) N 1s and (b) C 1s XPS spectrum of Fe$_{SA}$@NC and NC.

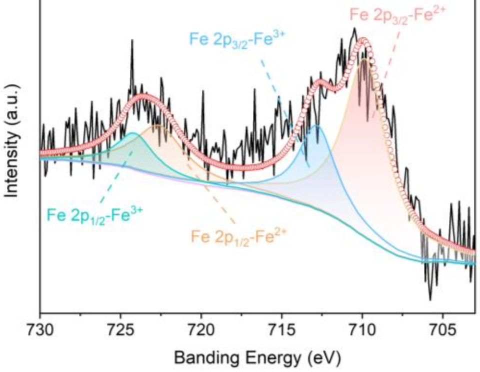

Figure S5. Fe 2p XPS spectrum of Fe$_{SA}$@NC.

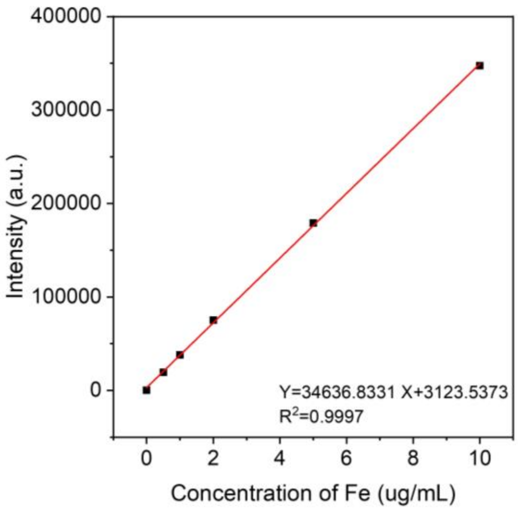

Figure S6. Calibration curve for determination of Fe content using ICP.

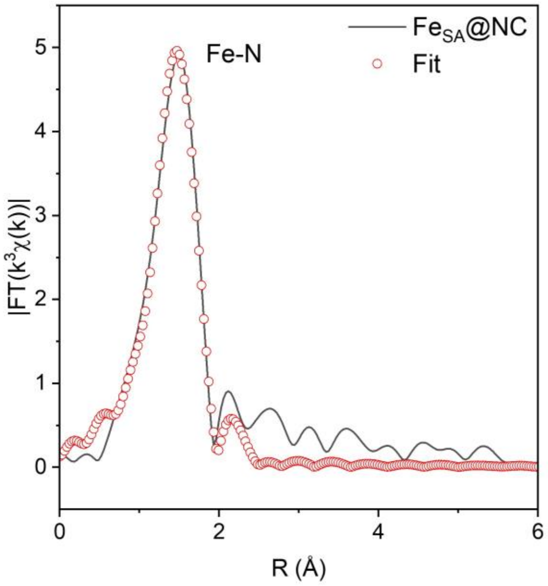

Figure S7. Experimental and FT-EXAFS fitting curves of Fe_{SA}@NC at Fe K-edge

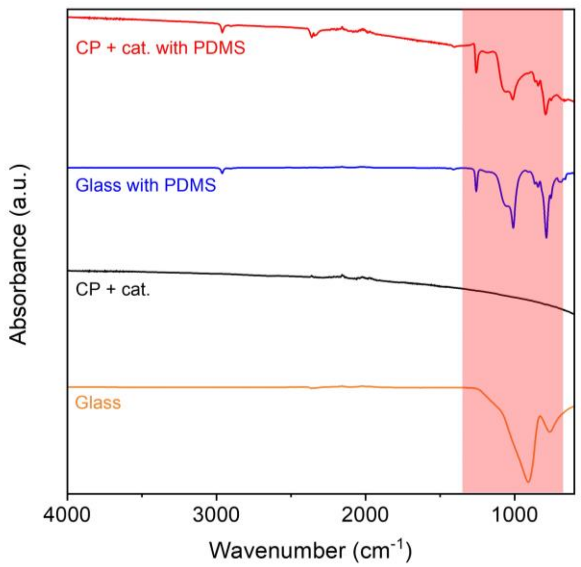

Figure S8. FTIR of different samples with or without PDMS.

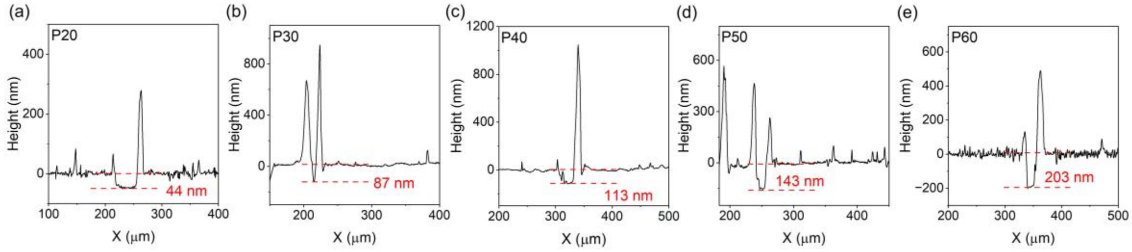

Figure S9. Step meter data of (a) Fe_{SA}@NC-P20, (b) Fe_{SA}@NC-P30, (c) Fe_{SA}@NC-P40, (d) Fe_{SA}@NC-P50 and (e) Fe_{SA}@NC-P60.

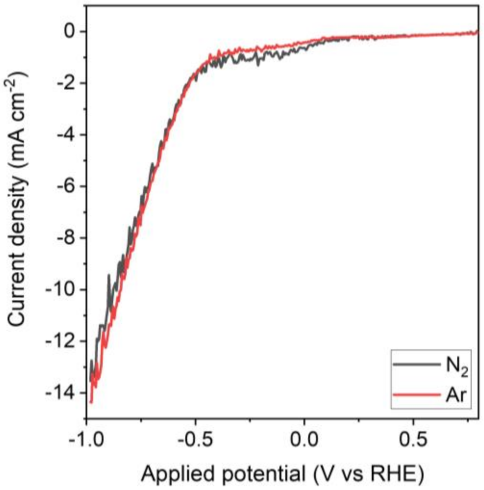

Figure S10. LSV curves of Fe_{SA}@NC in 0.1 M Na_2SO_4.

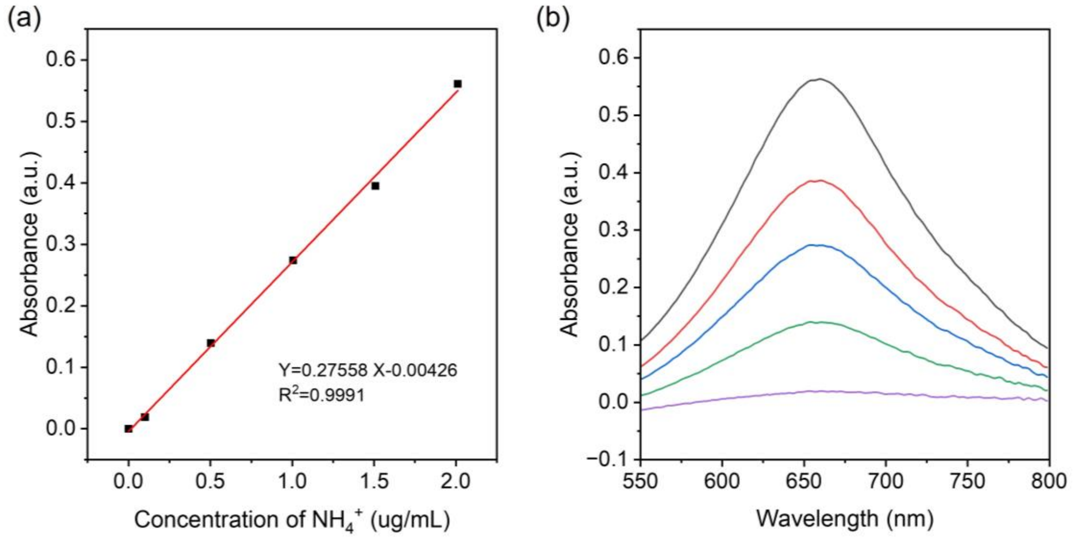

Figure S11. (a) Calibration curve for NH_4^+ detection using indophenol blue method. (b) Corresponding UV-Vis of calibration curve for NH_4^+.

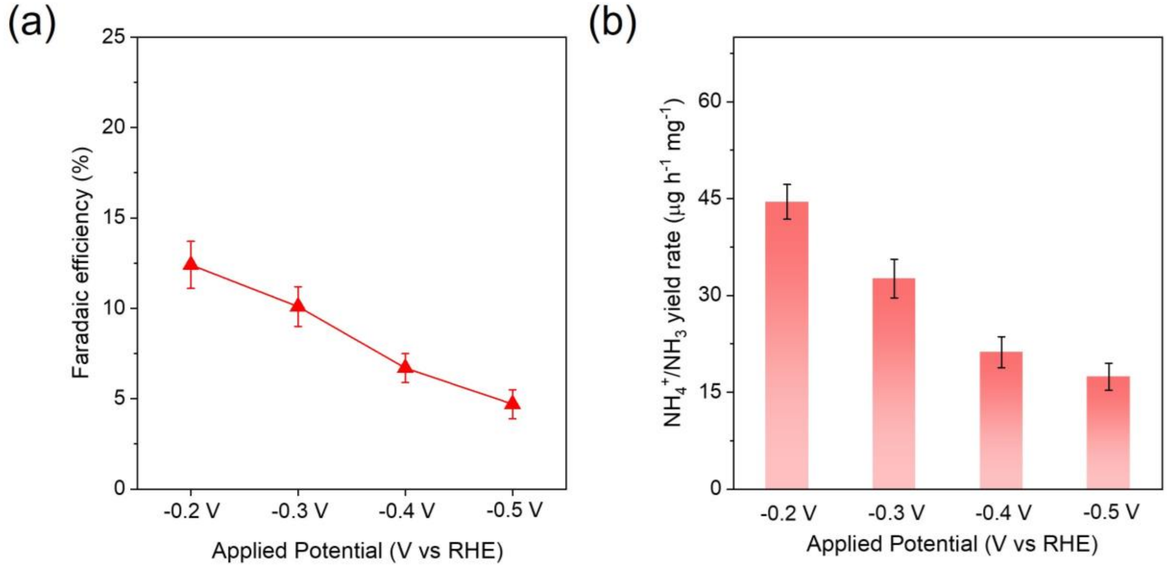

Figure S12. (a) Faradaic efficiency and (b) corresponding NH_3 yield rate of Fe_{SA}@NC in N_2 saturated 0.1 M Na_2SO_4.

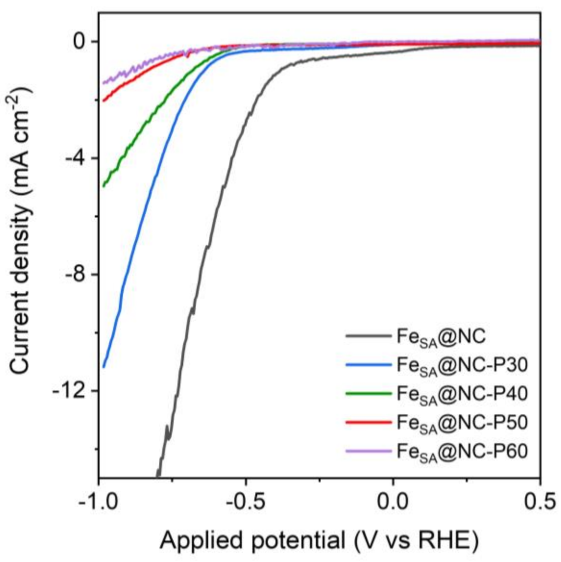

Figure S13. LSV curves of Fe_{SA}@NC with various thickness of PDMS membrane in N_2 saturated 0.1 M Na_2SO_4.

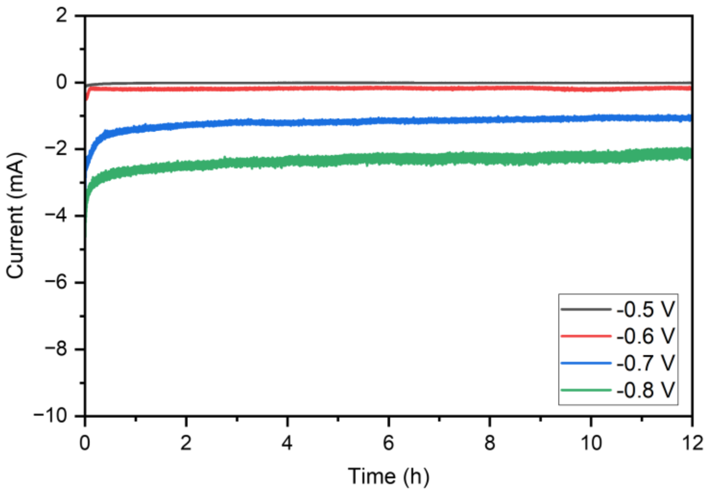

Figure S14. CA curves of Fe_{SA}@NC-P40 at various applied potentials.

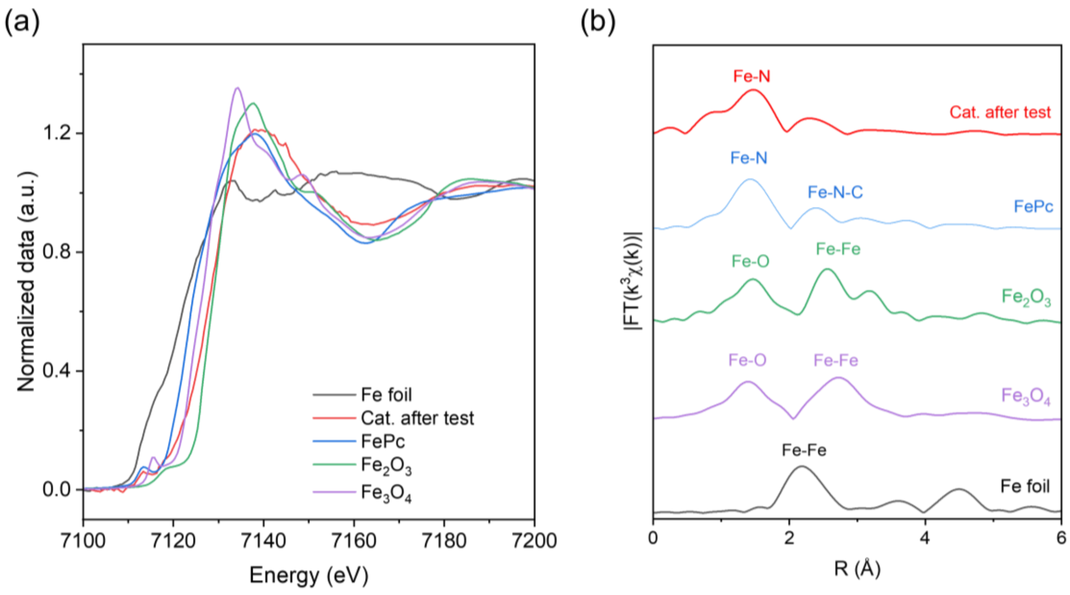

Figure S15. (a) Fe K-edge XANES and (b) FT EXAFS of as-prepared catalyst after stability test and reference samples.

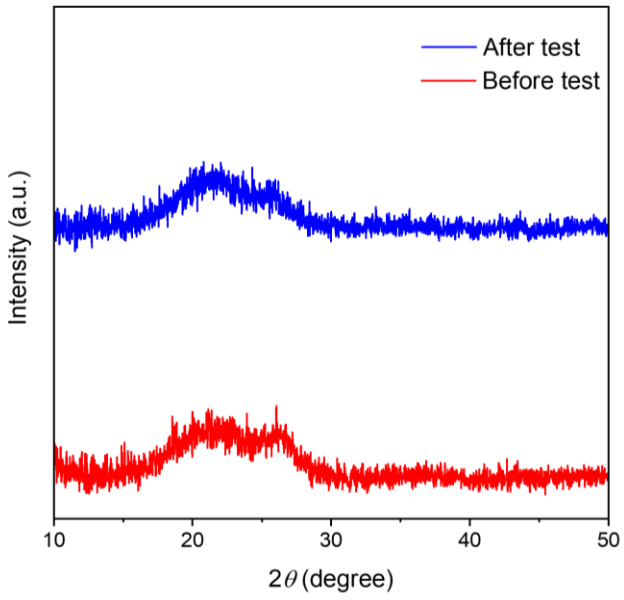

Figure S16. XRD of as-prepared catalyst before and after stability test.

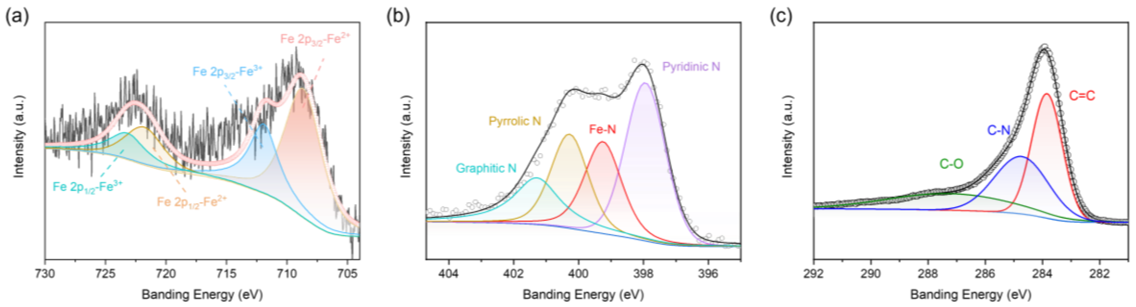

Figure S17. (a) Fe 2p, (b) N 1s and (c) C 1s XPS spectrum of as-prepared catalyst after stability test.

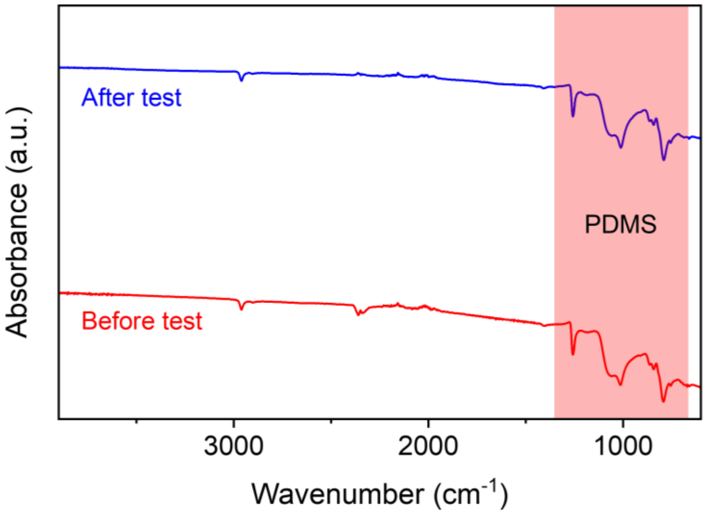

Figure S18. FTIR of as-prepared catalyst before and after long tern electrolysis.

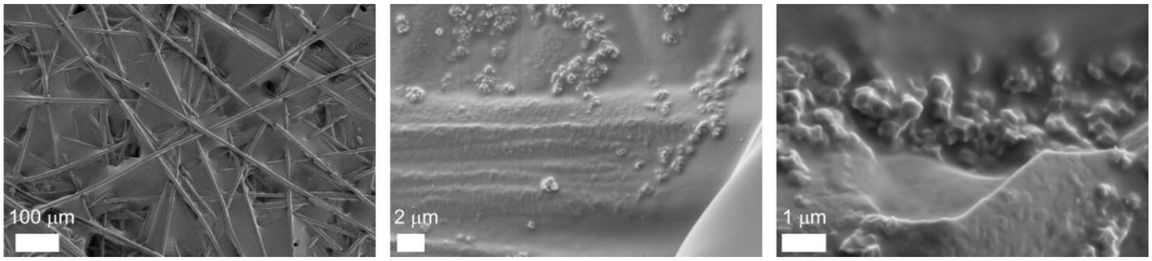

Figure S19. SEM images of Fe_{SA}@NC-P40 after electrolysis.

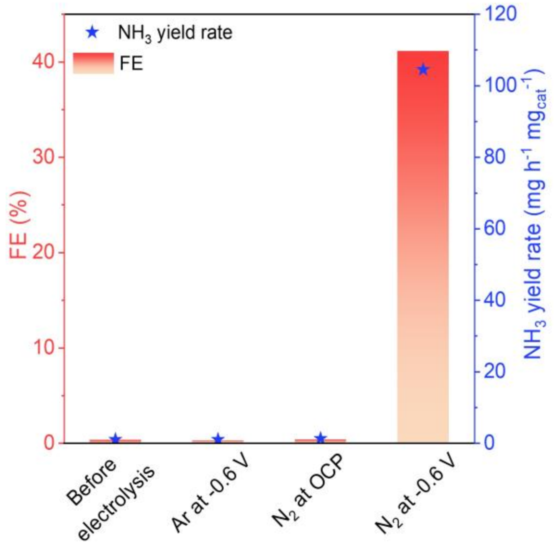

Figure S20. (a) NRR performance of Fe_{SA}@NC-P40 under different catalytic conditions.

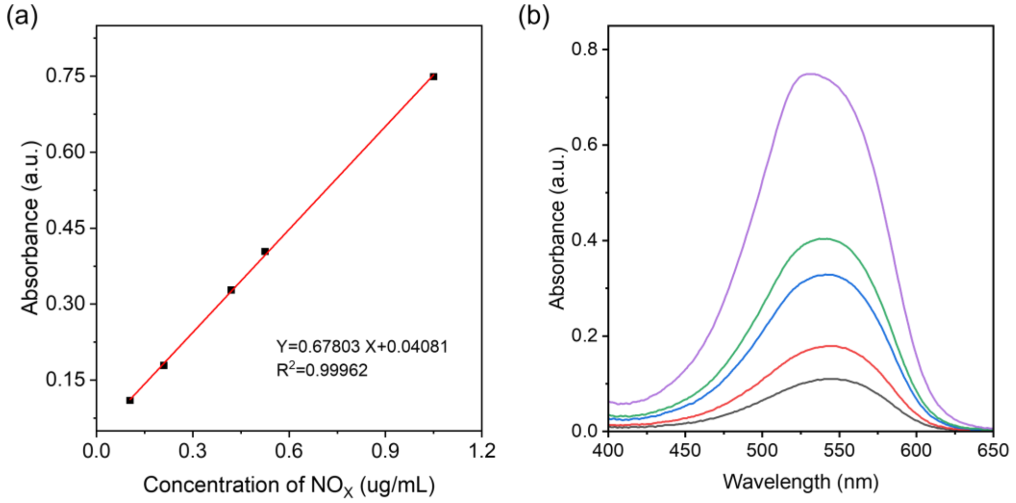

Figure S21. (a) Calibration curve for NO_x detection using *N*-(-1-naphthyl)-ethylenediamine dihydrochloride method. (b) Corresponding UV-Vis of calibration curve for NO_x.

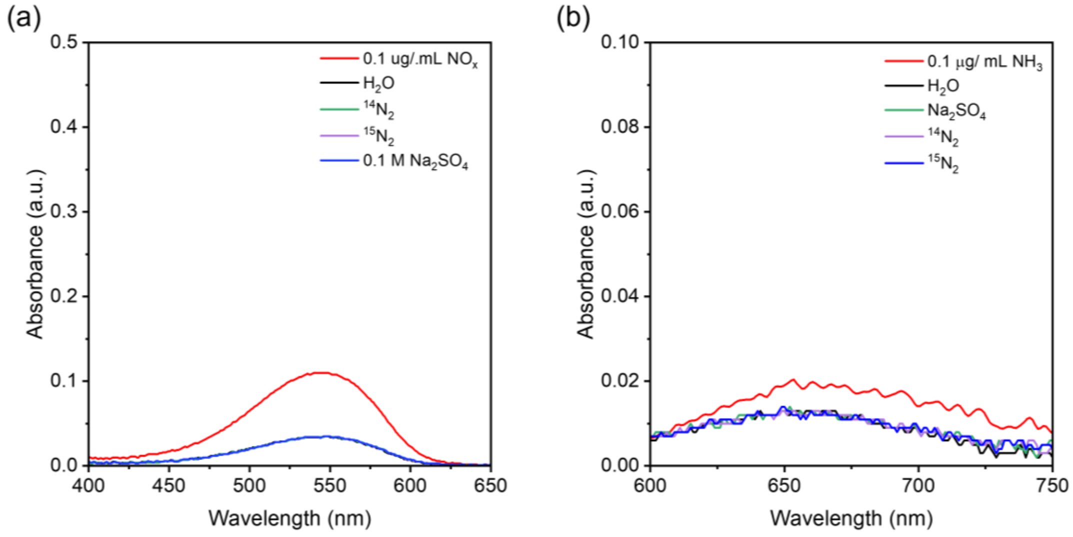

Figure S22. Detection of (a) NO_x and (b) NH_3 contaminates in N_2 feeding gas and electrolyte.

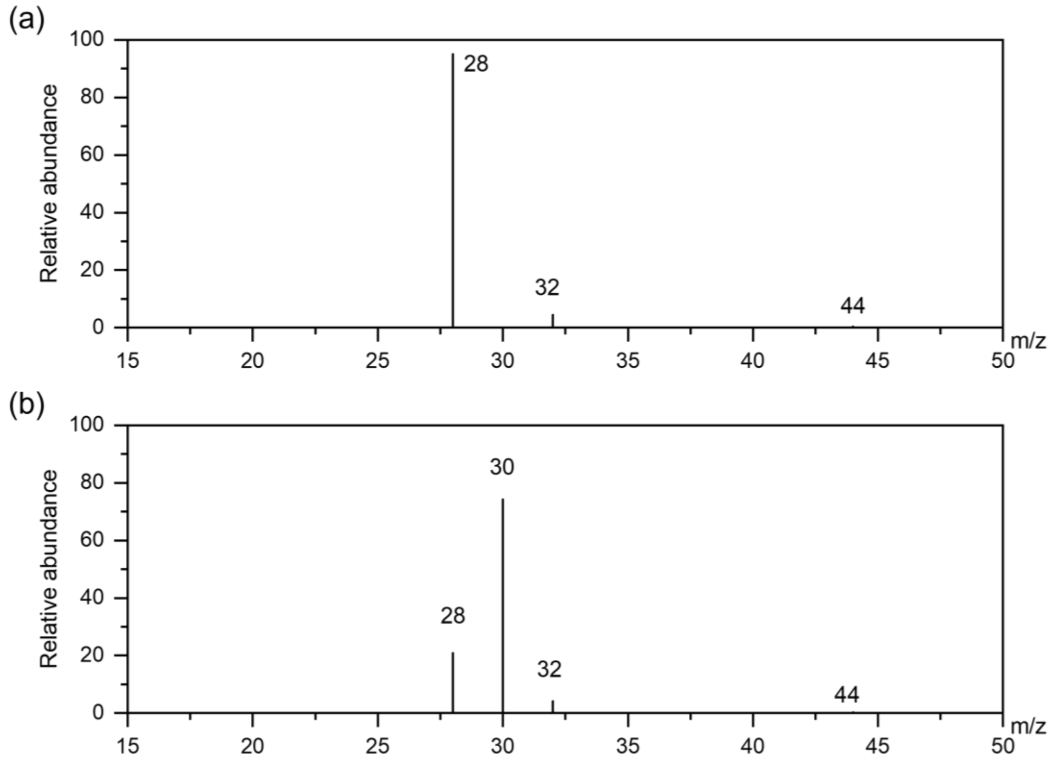

Figure S23. MS spectrum of (a) $^{14}N_2$ and (b) $^{15}N_2$ feeding gas.

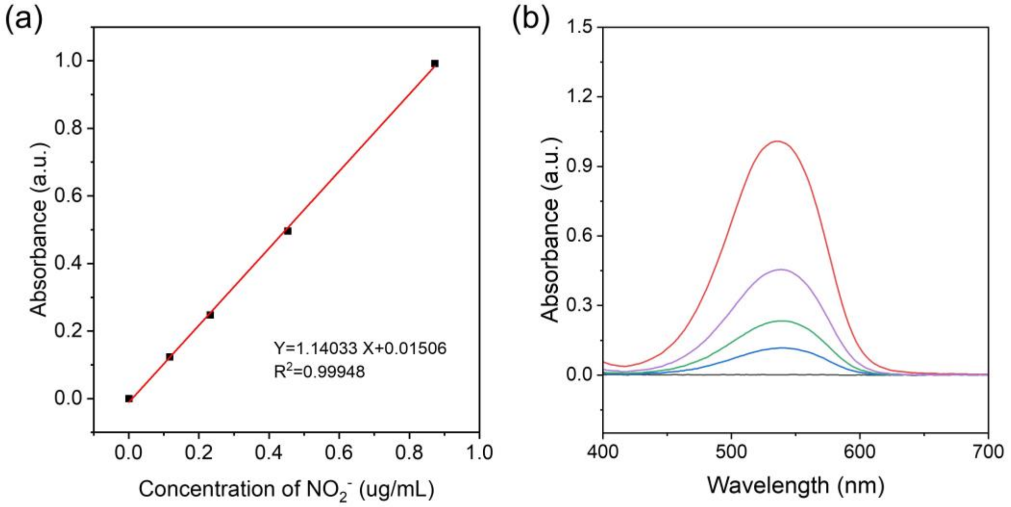

Figure S24. (a) Calibration curve for NO_2^-. (b) Corresponding UV-Vis spectrum for calibration curve.

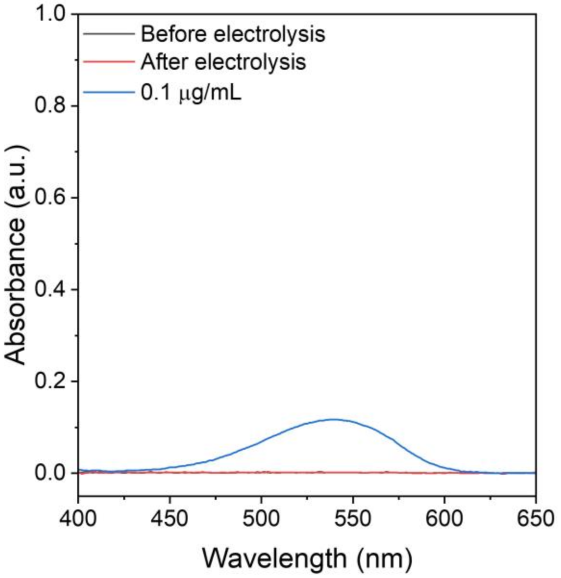

Figure S25. NO_2^- detection before and after electrolysis.

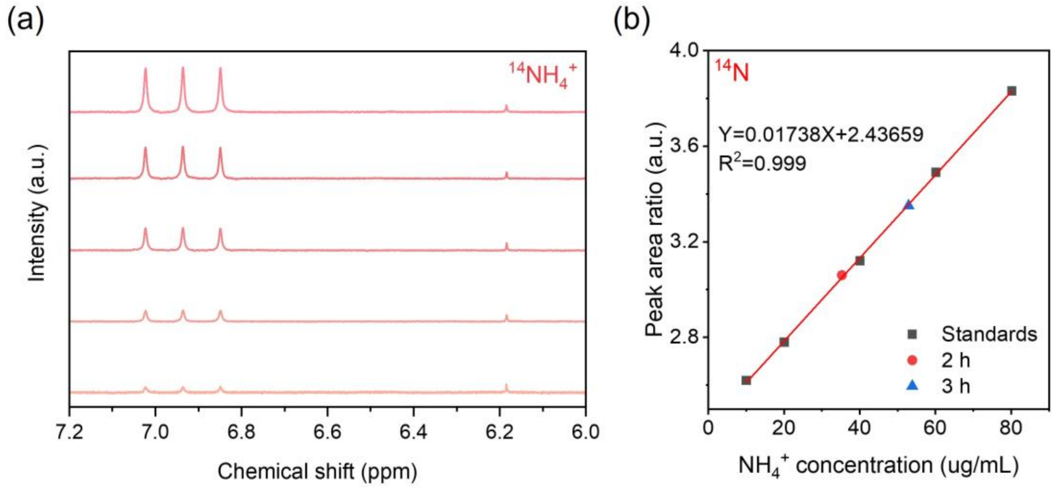

Figure S26. (a) ^{1}H NMR spectrum of $^{14}NH_4^+$ standards. (b) Calibration curve of NH_4^+ concentration versus peak area ratio for $^{14}NH_4^+$.

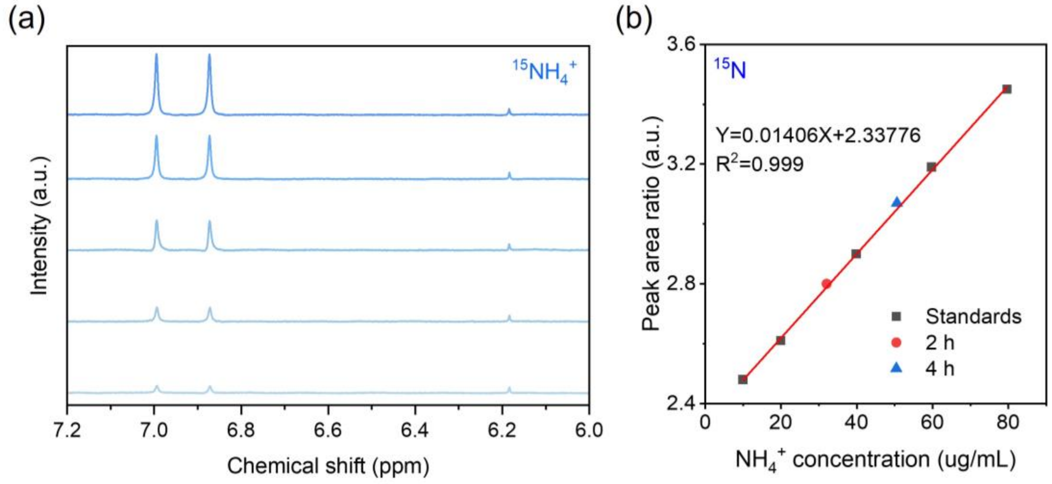

Figure S27. (a) ^{1}H NMR spectrum of $^{15}NH_4^+$ standards. (b) Calibration curve of NH_4^+ concentration versus peak area ratio for $^{15}NH_4^+$.

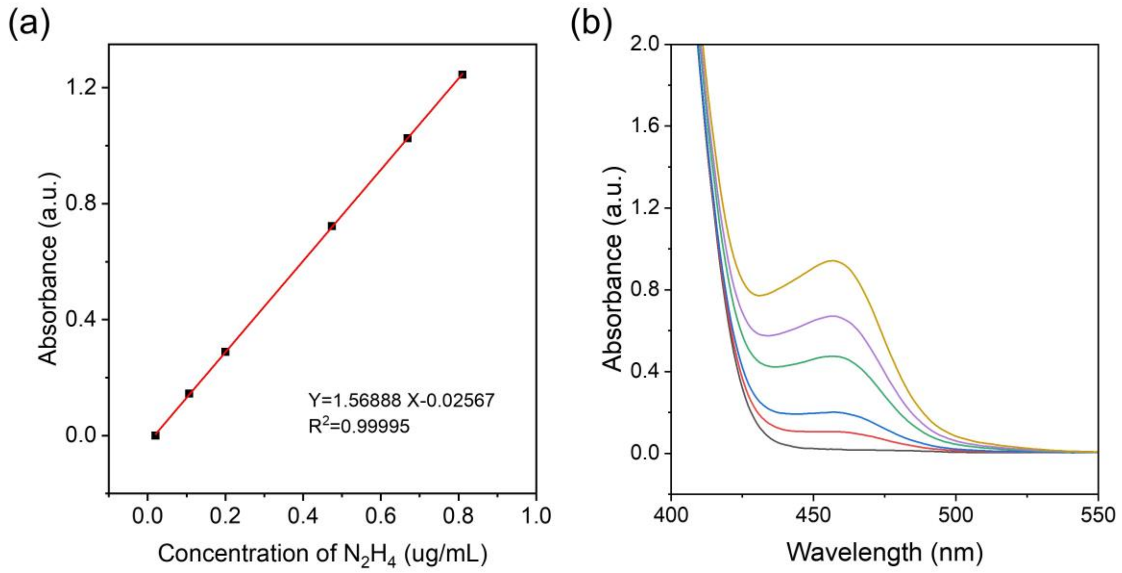

Figure S28. (a) Calibration curve for N_2H_4. (b) Corresponding UV-Vis spectrum for calibration curve.

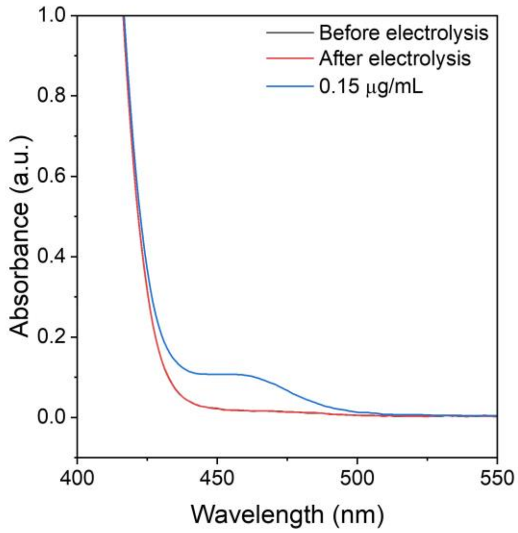

Figure S29. N_2H_4 detection before and after electrolysis.

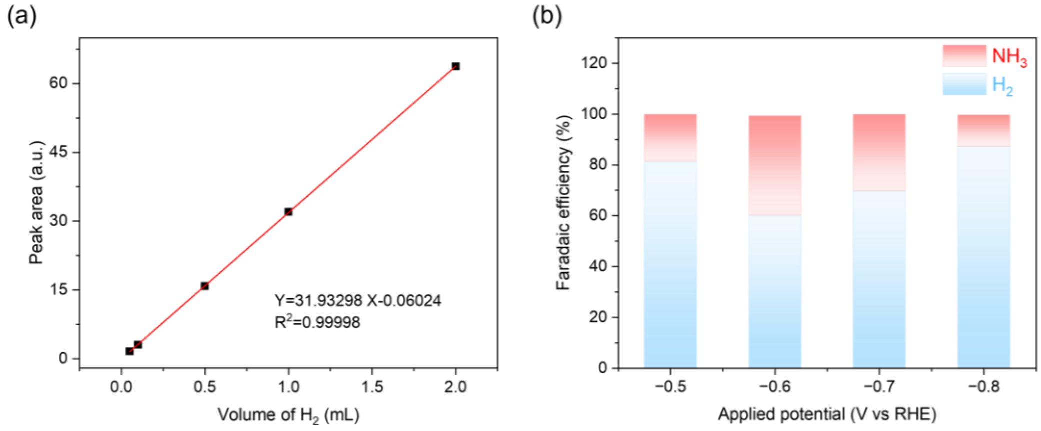

Figure S30. (a) Calibration curve of H_2 detection via GC. (b) Total Faradaic efficiency of Fe_{SA}@NC-P40 at various applied potential.

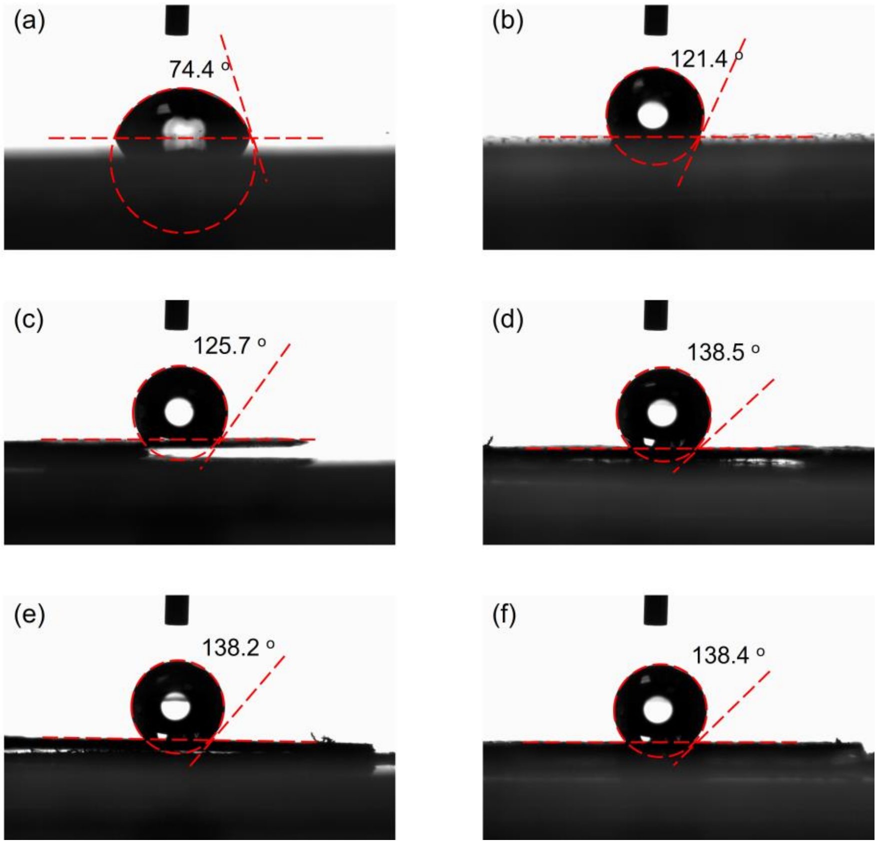

Figure S31. Optical images of contact angle of water over (a) glass, (b) glass with PDMS, (c) WE without PDMS, (d) Fe_{SA}@NC-P30, (e) Fe_{SA}@NC-P40 and (f) Fe_{SA}@NC-P50.

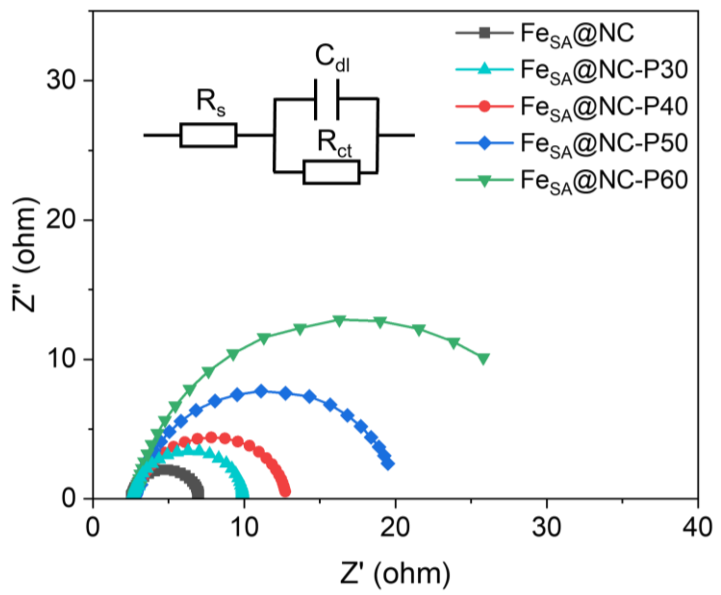

Figure S32. Nyquist plots of as-prepared samples and equivalent circuit. R_s and R_{ct} correspond to the resistances of the solution and charge transfer, C_{dl} to capacitance, respectively.

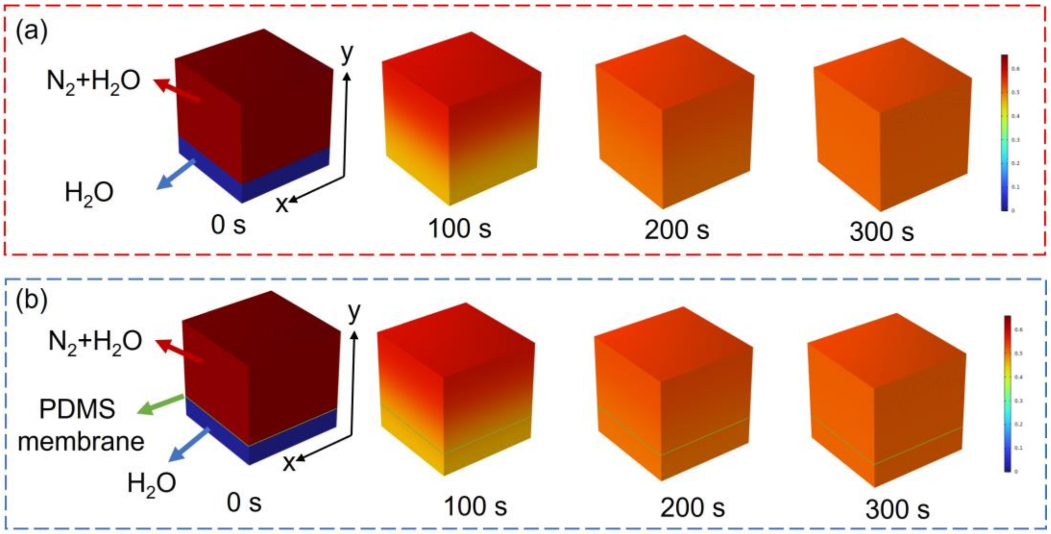

Figure S33. (a) Snapshots of N_2 diffusion behavior in a box with pure water at the bottom. (b) Snapshots of N_2 diffusion behavior in a box which is separated by PDMS membrane.

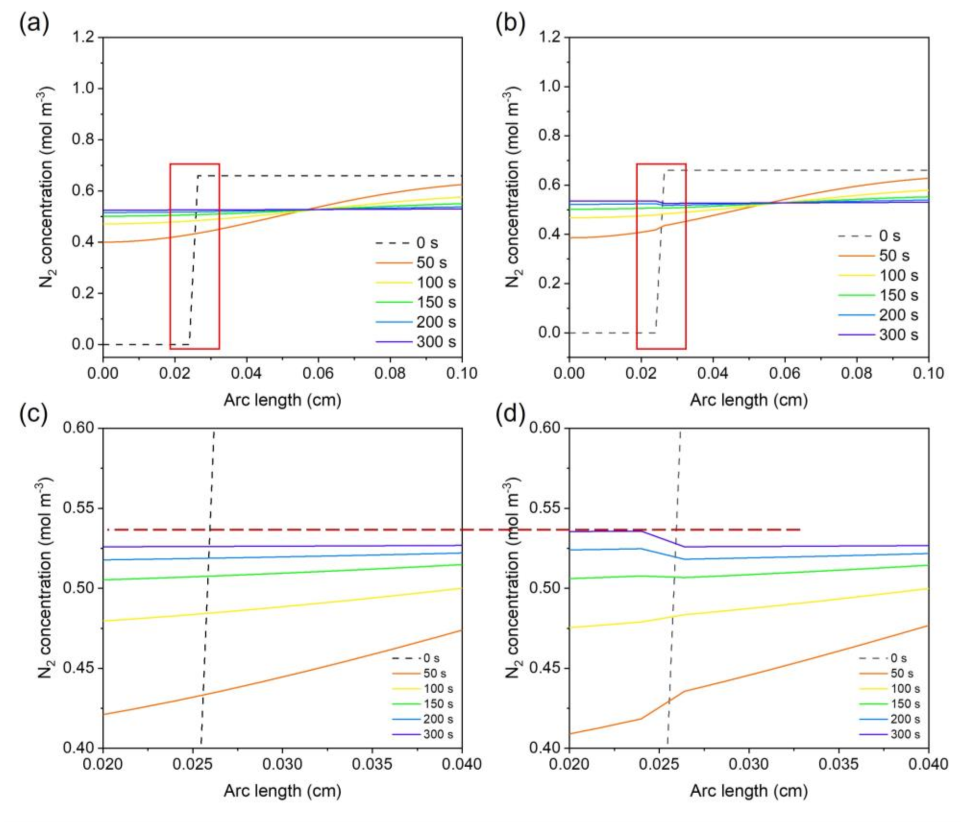

Figure S34. N_2 concentration along with y-direction of FES of (a) pure water at bottom, and of (b) separated by PDMS membrane. (c, d) Corresponding enlarged views of red rectangles in (a) and (b), respectively.

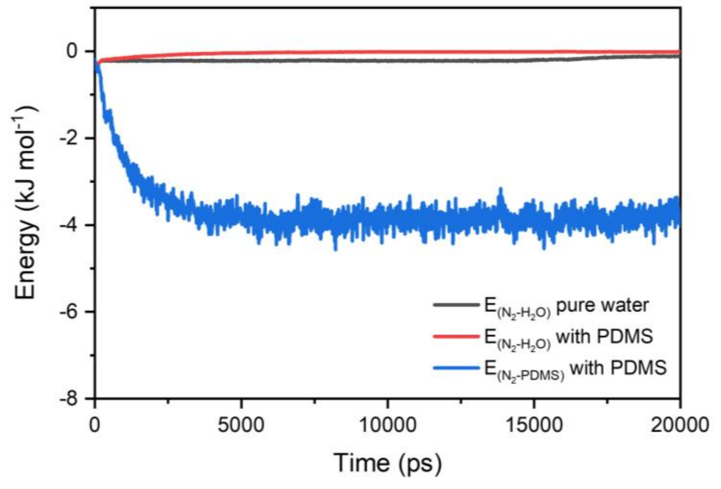

Figure S35. The van der Waals' force among N_2, H_2O and PDMS during MD simulation in different systems.

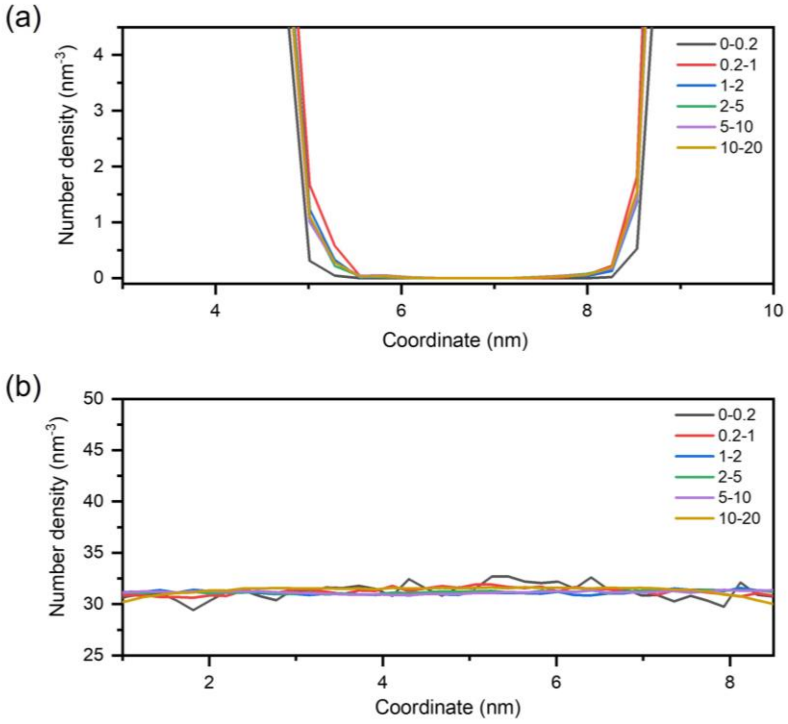

Figure S36. H_2O distribution of MD simulation (a) with and (b) without PDMS membrane.

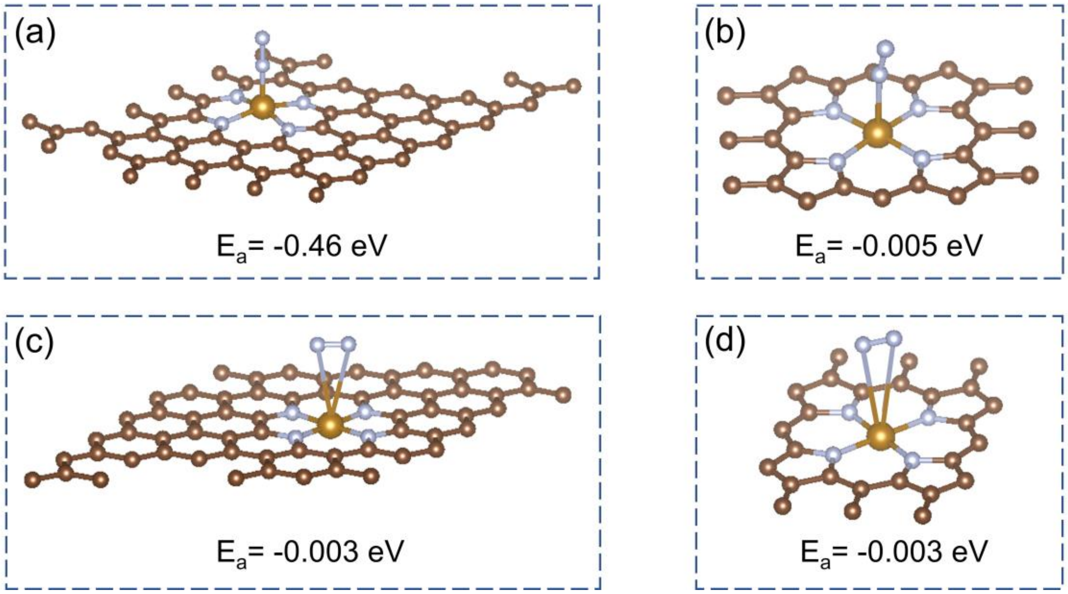

Figure S37. N_2 adsorbed on (a) FeN_4-pyridinic N or (b) FeN_4-pyrrolic N via end-on mode. N_2 adsorbed on (c) FeN_4-pyridinic N or (d) FeN_4-pyrrolic N via side-on mode. Gray circles for N atoms, brown circles for C atoms and yellow circles for Fe atoms.

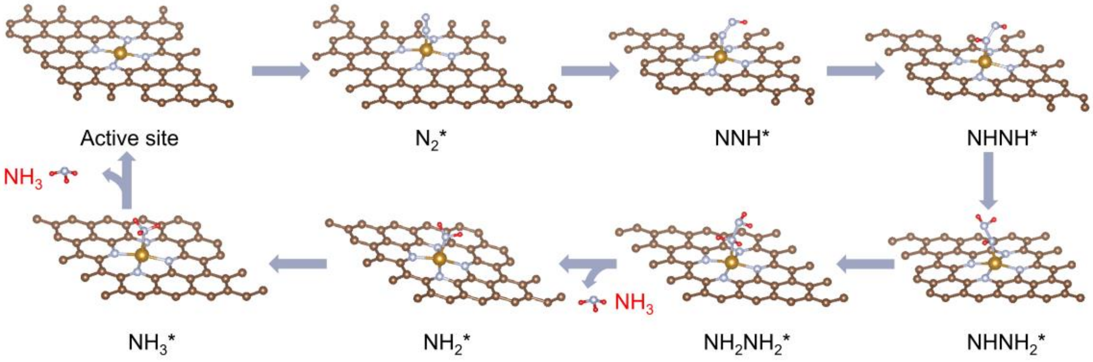

Figure S38. NRR on FeN_4-pyridinic N via alternating pathway. Gray circle for N atoms, brown circles for C atoms, red circles for H atoms and yellow circles for Fe atoms.

Table S1. Determination of Fe content in Fe_{SA}@NC.

Sample	Mass m_0 (g)	V_0 (mL)	Concentration of samples C_0 (mg/L)	Mass of element C_x (mg/kg)	wt (%)
1	0.0095	25	2.581	67934.2	6.79%
2	0.0095	25	2.595	68288.0	6.83%

Table S2. EXAFS fitting parameters at the Fe *K*-edge for various samples.

Sample	Shell	*CN*	*R*(Å)	σ^2(Å^2)	ΔE_0(eV)
Fe foil	Fe-Fe	8	2.475±0.004	0.0046±0.0016	7.2±0.9
	Fe-Fe	6	2.852±0.005	0.0071±0.0009	
FePc	Fe-N	4.0±0.1	1.979±0.014	0.0054±0.0023	9.3±2.1
	Fe-N-C	6.3±0.3	2.951±0.020		5.2±2.2
Fe_2O_3	Fe-O	6.0±0.6	1.962±0.023	0.0134±0.0033	-4.4±3.5
	Fe-Fe	3.9±0.8	2.964±0.013	0.0062±0.0015	2.0±2.2
	Fe-Fe	2.9±0.8	3.390±0.018		
	Fe-Fe	3.9±1.2	3.698±0.015		
Fe_3O_4	Fe-O	5.8±0.2	1.982±0.017	0.0066±0.0026	0.4±2.3
	Fe-Fe	3.8±0.9	2.981±0.005	0.0108±0.0019	0.9±2.5
	Fe-Fe	7.3±0.7	3.479±0.003		
Fe_{SA}@NC	Fe-N	3.7±0.3	1.990±0.016	0.0125±0.0027	3.3±2.0

Table S3. Comparison of electrochemical NRR performance of Fe_{SA}@NC-P40 with recently reported metal-based catalysts.

Catalyst	FE (%)	Yield rate (ug h^{-1} mg^{-1})	Ref
Fe_{SA}@NC	39.4	109.2	This work
FeOOH QDs-GS	14.6	27.3	Nano Res. 2020, 13, 209–214.
FePc-CNT	22.2	21.7	ACS Catal. 2022, 9, 5502–5509
ISAS-Fe/NC	18.6	62.9	Nano Energy 2019, 61, 420–427.
FeP_2-rGO	21.99	35.29	Chem. Commun. 2020, 56, 731–734.
FePc/C	10.5	137.95	ACS Catal. 2019, 9, 7311–7317.
NiO/G	7.8	18.6	ACS Appl. Energy Mater. 2019, 2, 2288–2295.
Cu NPS-rGO	15.32	24.58	J. Power Sources 2020, 448, 227417.
CuO/rGO	3.9	11.01	ChemCatChem 2019, 11, 1441–1447.
Cu-doped CeO_2	19.1	32.44	Chem. Commun. 2019, 55, 2952–2955.
Fe-Cu clusters	34	9.9	Adv. Mater. 2020, 32, 2004382.
$PdFe_1$	37.8	111.9	Angew. Chem., Int. Ed. 2022, 134, e2022059.
Fe_{SA}-N/CNT	9.28	34.83	ACS Catal. 2019, 9, 336-344.
Fe_{SA}-TPPCl	16.76	18.28	Appl. Catal. B Environ. 2021, 285, 119794.
Fe_{SA}-NO-C	11.8	31.9	Angew. Chem., Int. Ed. 2021, 60, 9078–9085.
Fe_3C/Fe_3O_4@C-950	22.5	25.7	Angew. Chem., Int. Ed. 2023, e202304797
$FeCuS_x$	75.05	27.4	Angew. Chem. Int. Ed. 2022, 61, e2022095.
PdCu/C-350	11.5	35.7	Angew. Chem., Int. Ed. 2020, 132,2671–2675.
CuAu@Cu	24.1	33.9	Appl. Catal. B Environ. 2021
Pd_{SA}-Cu	24.8	69.2	Angew. Chem. Int. Ed. 2020, 59, 2-8.
CN-Pd-NME	80.1	58.3	Adv. Funct. Mater. 2022, 32, 2109422.
Au-Fe_3O_4	10.54	21.42	Adv. Funct. Mater. 2020, 30, 1906579.
NC/Bi SAs/TiN/CC.	24.6	76.15	Adv. Sci. 2022, 9, 2104245
Sn_{SA}-ADP	26.8	28.3	Angew. Chem.Int. Ed.2023,62, e2022174.
Mo/VO_2	32.4	190.1	Adv. Energy Mater. 2023, 2203032.
COF-Fe/MXene	43.1	41.8	Adv. Sci. 2023, 10, 2206933
MoO_{2+x}	22.1	3.95	Adv. Sci. 2022, 9, 2104857
$PdFe_3$ nano-alloy	22.8	29.07	Adv. Energy Mater. 2023, 2303558
MoS_2-7H	14.68	66.74	Adv. Funct. Mater. 2023, 33, 2302501
Sb SA/N-$Ti_3C_2T_x$	41.2	108.3	ACS Nano 2023, 17, 21838−21849

IrTe alloy	11.2	34.6	J. Am. Chem. Soc. 2023, 145, 6079−6086
Fe−TiO_2	40.4	30.9	ACS Sustainable Chem. Eng. 2023, 11, 12345
Mo/BCN	13.27	37.67	ACS Catal. 2022, 12, 7655−7663
NH_x-gCN	27.00	56.38	Chem. Eng. J. 2023, 452, 139606
mRhTe/CP	20.4	41.7	J. Mater. Chem. A, 2023, 11, 17479
WSeS/WSe_2	40.2	15.96	Adv. Energy Mater. 2023, 13, 2301979
Ru/TiO_2	40.7	10.4	Chem. Catal. 2022, 2, 1764–1774
D-BP_{ene}	26	70	Angew. Chem. Int. Ed. 2023, 62, e202302124
Ag_4Ni_2 NCs	78.97	23.32	Adv. Funct. Mater. 2022, 32, 2202820
FeCoMOF-P_2W_{18}	31.56	47.04	Adv. Funct. Mater. 2023, 33, 2214495

Table S4. Calculation of turn over number per mole of catalyst and turn over frequency.

Catalysts	Amount of Fe single atom (10^{-3} μmol)	Amount of generated NH_3 (μmol)	TON per mole catalyst	TOF (h^{-1})
Fe_{SA}@NC	8.57	2.50	292	24
Fe_{SA}@NC-P30	8.57	3.34	390	32
Fe_{SA}@NC-P40	8.57	6.17	720	60
Fe_{SA}@NC-P50	8.57	5.15	601	50
Fe_{SA}@NC-P60	8.57	2.41	281	23

Table S5. Mass difference of H_2O evaporation with different thickness of PDMS.

Sample	Area of PMDS film (cm^2)	Initia Mass (g)	Mass after 24 h (g)	Mass difference (g)	Rate of H_2O evaporation (μmol h^{-1} cm^{-2})
P30	63.585	72.606	66.710	5.896	214.651
P40	63.585	72.381	66.559	5.822	211.972
P50	63.585	72.936	67.148	5.788	210.712

Table S6. Gibbs free energy (eV) for DFT calculations of NRR on FeN_4 in Fe_{SA}@NC.

	ΔE(DFT)	ΔZPE	TΔS	ΔG
slab	-446.2208836	-	-	-0
slab-N_2	-463.2893655	0.221904501	0.142680462	0.0594957
slab-N_2H	-465.6618746	0.461644039	0.228116799	1.285509322
slab-NNH_2	-469.3208003	0.81589295	0.134154273	1.519014481
slab-NNH_3	-471.6415838	1.1172406	0.250835778	2.827116618
slab-N	-452.6994918	0.084400412	0.063276739	2.066892233
slab-NH	-456.8295475	0.357151434	0.082342292	1.634741484
slab-NH_2	-461.8572999	0.662501325	0.117389926	0.321510823
slab-NH_3	-466.0473542	1.046687854	0.169945031	-0.092692571
slab	-446.2208836	-	-	-0
slab-N_2	-463.2893655	0.221904501	0.142680462	0.0594957
slab-N_2H	-465.6618746	0.461644039	0.228116799	1.285509322
slab-NHNH	-469.370469	0.821936338	0.175648888	1.433894574
slab-$NHNH_2$	-472.9905343	1.140867727	0.193959708	1.558669296
slab-NH_2NH_2	-476.8489243	1.4929538	0.158043951	1.532500626
slab-NH_2NH_3	-481.4776016	1.616197759	0.336112741	0.293217987
slab-NH_2	-461.8572999	0.662501325	0.117389926	0.321510823
slab-NH_3	-466.0473542	1.046687854	0.169945031	-0.092692571
N_2	-16.60851188	0.153858279	0.5941	-
H_2	-6.75219026	0.267751296	0.404	-

Reference

[1] Z. Wang, X. Jin, C. Zhu, Y. Liu, H. Tan, R. Ku, Y. Zhang, L. Zhou, Z. Liu, S. J. Hwang, H. J. Fan, *Adv. Mater.* **2021**, 33, e2104718.

[2] M. Wang, S. Liu, H. Ji, T. Yang, T. Qian, C. Yan, *Nat. Comm.* **2021**, 12, 3198.

[3] G. Kresse, J. Furthmüller, *Comp. Mater. Sci.* **1996**, 6, 15-50.

[4] J. P. Perdew, K. Burke, M. Ernzerhof, *Phys. Rev. L.* **1996**, 77, 3865-386.

[5] Computational Chemistry Comparison and Benchmark Database. http://cccbdb.nist.gov/

[6] M. J. Abraham, T. Murtola, R. Schulz, S. Páll, J. C. Smith, B. Hess, E. Lindahl, *SoftwareX* **2015**, 1, 19-25.

[7] L. Martínez, R. Andrade, E. G. Birgin, J. M. Martínez, *J. Comput. Chem.* **2009**, 30, 2157.

[8] W. L. Jorgensen; J. Chandrasekhar; J. D. Madura; R. W. Impey; M. L. Klein, *J. Chem. Phys.* **1983**, 79, 926.

[9] J. Wang, R. M. Wolf, J. W. Caldwell, P. A. Kollman, D. A. Case, *J. Comput. Chem.* **2004**, 25, 1157.

[10] J. J. Potoff, J. I. Siepmann, *AIChE J.* **2001**, 47, 1676-1682.
